# Casein genotypes associate with baseline and dynamic regression components of seminal quality in Murciano-Granadina bucks

**DOI:** 10.1038/s41598-026-43947-1

**Published:** 2026-04-15

**Authors:** María Peláez Caro, Ander Arando Arbulu, José Manuel León Jurado, Juan Vicente Delgado Bermejo, Javier Fernández Álvarez, Francisco Javier Navas González

**Affiliations:** 1 Department of Genetics, Faculty of Veterinary Medicine, University of Córdoba, Córdoba, Spain; 2 National Association of Breeders of Murciano-Granadina Goat Breed, Granada, Spain; 3 Centro Agropecuario Provincial de la Diputación de Córdoba, Córdoba, Spain

**Keywords:** Genomic selection, Nonlinear modelling, Sperm motility, Ejaculate volume, Membrane integrity, Genetics, Physiology

## Abstract

This study examined associations between αS1- and κ-casein genotypes and baseline and dynamic regression components of semen quality in Murciano-Granadina bucks using cubic regression models applied to 6,868 ejaculates collected over 10 years, with age at sperm collection as the time axis. Regression decomposition into baseline (b_0_), linear (b_1_), curvature (b_2_), and cubic tren (b_3_) coefficients enabled quantification of static trait levels and age-dependent dynamics, revealing substantial coefficient variability. For αS1-casein, genotype was associated with baseline progressive motility and total added semen volume (*p* < 0.05), with moderate to large discriminant effects for progressive motility (ηp^2^ = 0.277, 95% CI 0.03–0.48) and curvature-related motility and Acrosome Integrity/Intact acrosomes components (ηp^2^ = 0.212–0.245), indicating genotype-related differences in modeled semen quality rather than direct fertility effects. In contrast, κ-casein genotypes showed predominantly small to moderate effects, mainly related to ejaculate volume dynamics and baseline sperm concentration (ηp^2^ = 0.131–0.138), while most other components were negligible. Cubic terms captured subtle non-linear trajectories, particularly for sperm concentration and endosmosis, highlighting their relevance for long-term trait stability. Discriminant analyses indicated model-based separation of αS1–κ genotype combinations, with EE–AA (*n* = 1) and BE–AA (*n* = 3) aligning with higher sperm motility, concentration, and favorable membrane-integrity responses, whereas BB–BB (*n* = 3) aligned with higher semen volume and lower quality traits. Validation procedures were method-specific. CDA using leave-one-out cross-validation showed stronger discrimination for αS1-casein (74.03%) than κ-casein (64.94%), driven by perfect classification of the dominant BE genotype and substantial misclassification of rarer genotypes, whereas CHAID using 10-fold cross-validation showed moderate predictive performance with lower risk for αS1-casein (0.325, SE = 0.074; accuracy = 67.53%) than κ-casein (0.429, SE = 0.079; accuracy = 57.14%). Press’ Q statistics confirmed that classification accuracy for both genotypes exceeded chance expectation across methods (CDA: αS1-casein Q = 182.40, κ-casein Q = 34.60; CHAID: αS1-casein Q = 143.44, κ-casein Q = 19.64; *p* < 0.05), with consistently stronger discrimination for αS1-casein genotypes. Overall, baseline and dynamic regression components capture biologically relevant, exploratory associations between casein polymorphisms and semen quality dynamics, supporting their integration into genomics-informed reproductive management strategies.

**Supplementary Information:**

The online version contains supplementary material available at 10.1038/s41598-026-43947-1.

## Introduction

The exploration of the relationship between casein genotype and seminal quality in Murciano-Granadina goats is grounded in the broader context of livestock management and breeding programs. Murciano-Granadina goats, renowned for their prolificacy and adaptability, are a significant genetic resource for the dairy industry^1^. As genetic advancements become increasingly pivotal in optimizing livestock production, understanding the nuances of reproductive traits in this breed assumes paramount importance^2^.

Casein, a group of phosphoproteins found in milk, is known for its multifaceted functions. Beyond its traditional association with milk quality and composition, casein has been implicated in various physiological processes, including immune response modulation^3^ and reproductive physiology^4^. The intricate interaction between genetics and reproductive outcomes is especially relevant in Murciano-Granadina goats, where casein genotype and genes related to milk yield and udder traits may play a pivotal role in shaping semen characteristics, including volume and quality^5^. For example, Guan et al.^6^ investigated the molecular basis of lactation and identified genetic factors influencing milk yield and composition, which could offer insights into how milk-related genetic factors might indirectly affect seminal quality.

Semen, the vehicle for transmitting genetic material from males to females, is a crucial determinant of reproductive success^7^. Sperm motility, morphology, and concentration are essential factors conditioning quality^8^^,9^, and have a direct relationship with fertility outcomes^7^. Understanding how casein genotypes may impact these parameters is therefore key to uncovering new insights into the genetics of reproduction in Murciano-Granadina goats. 

Over the past two decades, global production of goat-derived products—including milk, cheese, and meat—has risen by 66%, largely driven by the genetic selection of highly productive bucks and does, with a strong focus on improving milk yield and composition^10^. Among Spanish native breeds, Murciano-Granadina goats have gained prominence as a valuable genetic resource due to their superior dairy traits, often exceeding international breeds such as Saanen and Anglo-Nubian in protein, fat, and total solids content^1,11,12^. To meet the growing demand for high-quality dairy products, breeder associations like CAPRIGRAN have implemented advanced selection programs that integrate genetic evaluation, artificial insemination, and genomic tools^13,14^. Within these programs, bucks are routinely genotyped for casein variants, as these genes play a central role in determining both the quantity and technological properties of milk proteins^15^. Among the four casein genes—CSN1S1, CSN2, CSN1S2, and CSN3—clustered on chromosome 6^16,17^, αS1-casein (CSN1S1) and κ-casein (CSN3) are of particular interest due to their pronounced effects on protein yield, milk composition, and cheese-making quality^18–21^. Functionally, αS1-casein contributes to calcium and phosphate binding, whereas κ-casein is critical for micelle stabilization and influences milk clotting and curd formation^22^.

Although the casein locus has been extensively studied for milk traits, emerging evidence indicates it may also influence male reproductive traits. Both αS1- and κ-casein genes are regulated by hormones such as prolactin, growth hormone, insulin-like growth factor 1 (IGF-1), and steroid hormones—many of which also govern testicular development, spermatogenesis, and accessory gland function. Variants in these genes can modify transcriptional activity and protein expression in response to these endocrine signals, suggesting possible pleiotropic effects beyond the mammary gland. For instance, αS1-casein genotypes differ in promoter responsiveness to prolactin and glucocorticoids^23^, which may indirectly modulate Leydig cell testosterone production, Sertoli cell activity, and spermatogenic efficiency. Likewise, κ-casein polymorphisms, by altering calcium binding and micelle dynamics, may influence systemic mineral–endocrine balance, which is critical for sperm motility, capacitation, and the acrosome reaction. Additionally, the casein genomic region overlaps with quantitative trait loci (QTLs) associated with fertility, growth, and metabolism^24^, highlighting potential linkage or pleiotropy coupling dairy traits with reproductive efficiency.

This functional overlap opens opportunities for indirect selection strategies. Since the effects of casein genotypes are well characterized in females—such as daughters, sisters, and dams—these data can provide predictive information for related males. If semen quality is partially modulated by casein-linked endocrine pathways, selecting favorable alleles in females could simultaneously enrich the male population for improved reproductive potential. Integrating this dual selection approach allows breeders to optimize both milk production and male fertility within a unified genetic framework, enhancing the efficiency and sustainability of breeding programs.

To date, evidence for the influence of casein genes on male reproductive traits remains limited. In goats, casein polymorphisms have primarily been linked to milk yield and composition, although CSN3 variants have also been associated with genetic values in bucks^21^. In cattle, lower casein percentages have been correlated with reduced fertility outcomes^4^. These findings suggest that casein variants may extend their effects beyond lactation, underscoring the need for systematic studies in goats.

The present study addresses this gap by applying cubic regression modelling to seminal quality traits in Murciano-Granadina bucks, focusing on αS1- and κ-casein genotypes. Cubic regression allows the partitioning of variation into baseline, linear, curvature (quadratic), and cubic trends, capturing both simple and complex patterns of change in seminal parameters. This approach enables the characterization of how specific genotypes influence non-linear dynamics in semen quality, providing actionable insights for integrating genetic markers of dairy performance and reproductive efficiency into breeding programs.

The aim of this study was to evaluate the influence of αS1- and κ-casein genotypes on seminal quality in Murciano-Granadina bucks using cubic regression, partitioning variation into baseline, linear, curvature, and cubic trends, to support genomics-informed breeding strategies.

## Results

### A priori assumptions

Preliminary analyses indicated departures from strict normality in several semen quality parameters. Although the Shapiro–Francia W′ test rejected the null hypothesis of normality (*p* < 0.05), this result was primarily driven by mild deviations in the distribution tails, associated with a very small proportion of extreme observations (< 1% of values). Visual inspection of Q–Q plots indicated that the central portion of the distributions closely followed normal expectations, supporting approximate normality of the data. Consequently, the observed departures were considered biologically and statistically negligible, and parametric analyses were performed at both analytical levels used in the study. Under these conditions, the data were considered sufficiently normal for the applied parametric and multivariate analyses.

Outlier screening using the ROUT method (false discovery rate Q = 1%) implemented in the Identify Outliers routine of GraphPad Prism version 9.0 (GraphPad Software, San Diego, CA, USA) did not identify any significant outliers. All observations were therefore retained for subsequent analyses.

Durbin–Watson (DW) statistics for all semen quality parameters were close to 2 (mean range 1.63–1.82), indicating no meaningful first-order autocorrelation in age-ordered residuals within bucks (Supplementary Table S1).

Levene’s test for homogeneity of variances of semen quality parameters across αs1- and κ-casein genotype groups indicated heteroscedasticity (*p* < 0.05); therefore, canonical discriminant analysis was used, which is robust to moderate departures from normality and variance homogeneity.

### Descriptive statistics

**Fig. 1:**
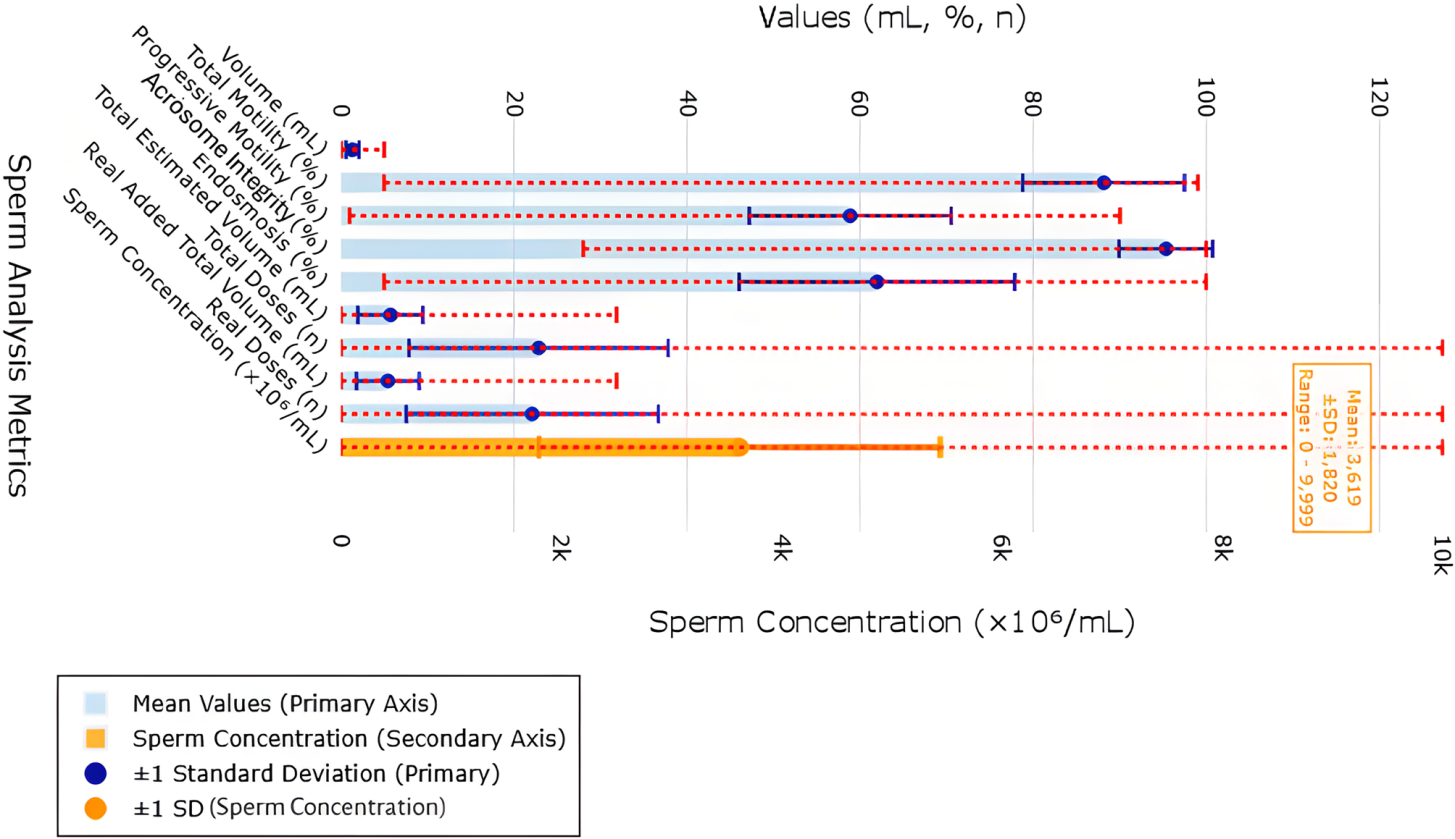
Summary of sperm analysis metrics showing mean values (blue bars), standard deviations (blue error markers), and sperm concentration (orange markers, secondary axis). Primary axis includes semen volume, motility, morphology, sperm count, and estimated/added volumes, while the secondary axis highlights sperm concentration (×10^6^/mL). Error bars indicate ± 1 SD. Canonical Discriminant Analysis.

Figure 1 presents the descriptive statistics for semen quality traits, highlighting substantial variability among samples. Ejaculate volume averaged 1.34 mL, with values ranging from 0 to 5.0 mL. Sperm concentration showed marked dispersion, with a mean of 3619 × 10^6^/mL and an extended range from 0 to 9999 × 10^6^/mL, indicating strong individual differences among bucks. Motility parameters were generally high, with total motility averaging 88.13% (SD = 9.35) and progressive motility 58.85% (SD = 11.67). Morphological integrity, reflected by a low proportion of abnormal spermatozoa, remained consistently high with a mean of 95.32%. By contrast, sperm viability (endosmosis, 61.95%) and production-related measures—including total sperm output (Total Estimated Volume, Real Added Total Volume) and ejaculate-level productivity (Total and Real Doses)—displayed broader ranges, emphasizing the heterogeneity of semen quality across the dataset.

### Variance inflation factors and multicollinearity management

Several seminal quality curve parameters exhibited severe multicollinearity during the initial stages of the VIF analysis, with tolerance values approaching zero and VIFs reaching extremely inflated levels (often > 70,000), indicating substantial redundancy among predictors. To address this issue, an iterative stepwise reduction procedure was applied, whereby a single parameter was removed at each round based on its contribution to collinearity.

Specifically, the following parameters were sequentially discarded: Constant Real Doses (Round 1), b_2_ Real Doses (Round 2), b_2_ Total Doses (Round 3), Constant Total Doses (Round 4), b_3_ Total Motility (Round 5), b_3_ Total Doses (Round 6), b_3_ Real Doses (Round 7), b_3_ Sperm Concentration (Round 8), b_3_ Progressive Motility (Round 9), Constant Volume (Round 10), Constant Total Estimated Volume (Round 11), b_3_ Total Real Added Volume (Round 12), b_3_ Volume (Round 13), b_3_ Acrosome Integrity/Intact acrosomes (Round 14), Constant Total Motility (Round 15), b_2_ Sperm Concentration (Round 16), b_2_ Total Real Added Volume (Round 17), b_3_ Total Estimated Volume (Round 18), b_1_ Endosmosis (Round 19), and b_2_ Total Motility (Round 20).

After 21 rounds of collinearity diagnostics, the final model retained predictors representing both baseline (constant) and dynamic (polynomial) components of semen quality traits. Baseline components included sperm concentration, progressive motility, total real added volume, endosmosis, and Acrosome Integrity/Intact acrosomes. Dynamic components comprised second-order (b_2_) terms for total estimated volume, progressive motility, Acrosome Integrity/Intact acrosomes, and volume, as well as a third-order (b_3_) term for endosmosis. The complete list of retained parameters, together with their corresponding collinearity diagnostics (tolerance and VIF values), is provided in Supplementary Table S2.

Across successive iterations, R^2^ values declined, tolerance values increased, and VIFs were progressively reduced to acceptable levels (< 5), indicating effective mitigation of multicollinearity. This refinement ensured that the remaining seminal quality parameters could be interpreted reliably in subsequent regression analyses, with their effects more clearly distinguished and less confounded by redundancy among explanatory terms (Supplementary Table S2).

Model stability was assessed through convergence of multicollinearity diagnostics across successive rounds of predictor reduction. Initial models exhibited extreme numerical instability, with variance inflation factors (VIFs) exceeding 70,000, reflecting severe redundancy among polynomial components and closely related seminal traits. With each deletion step, maximum VIF values decreased monotonically and converged below the conservative threshold of VIF ≤ 5 in the final model (Round 21), with no subsequent rebound. This progressive and order-of-magnitude reduction demonstrates effective resolution of multicollinearity and stabilization of the predictor space. Together with acceptable tolerance values and consistent canonical structures observed across late reduction rounds, these results indicate that the final model is numerically stable and well-conditioned, allowing reliable estimation and interpretation of discriminant functions.

**Fig. 2:**
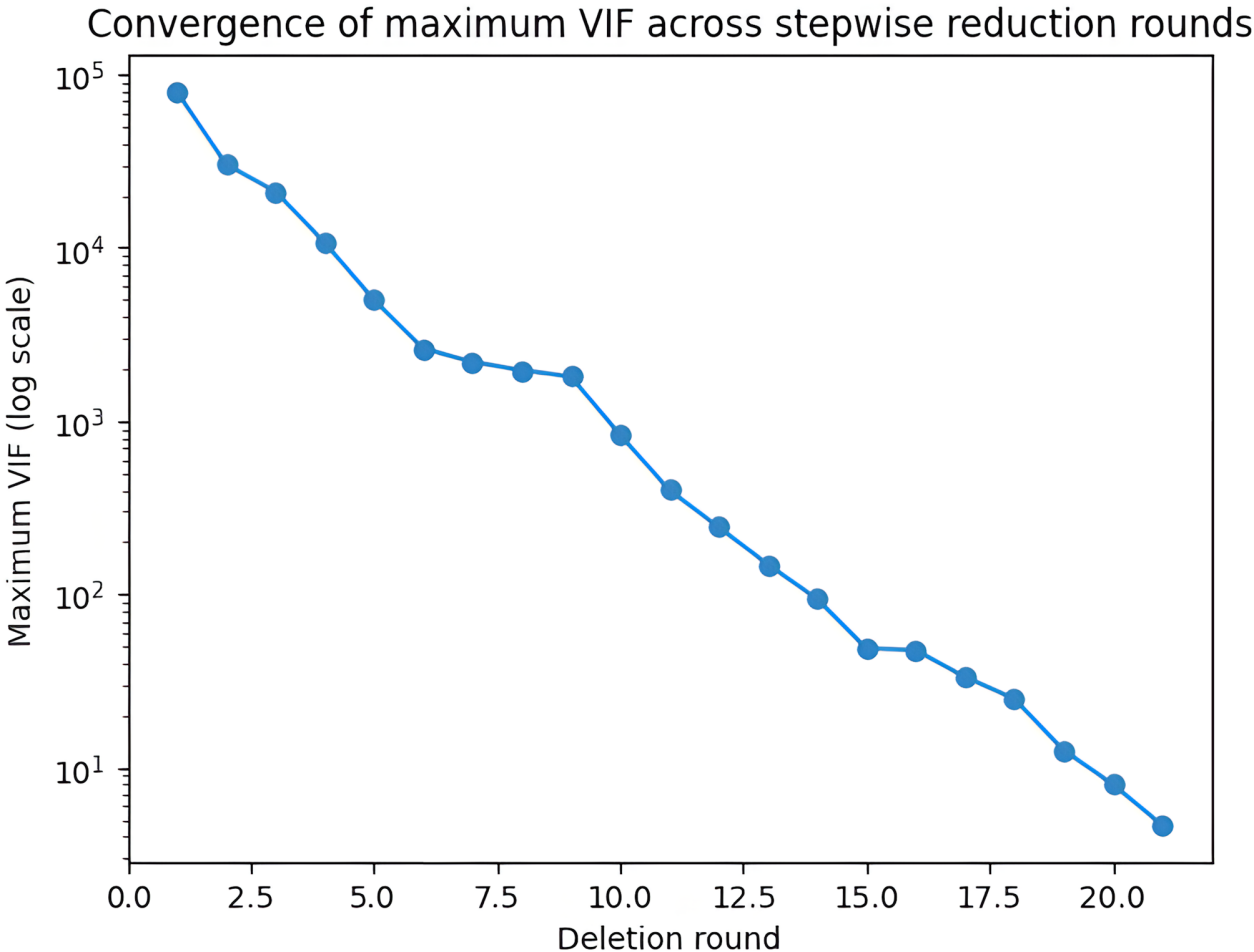
Evolution of maximum variance inflation factor (VIF) values across successive rounds of stepwise predictor removal. Maximum VIF values decreased monotonically from > 70,000 in the initial model to < 5 in the final model (Round 21), indicating effective resolution of extreme multicollinearity and convergence toward a numerically well-conditioned predictor set.

The VIF convergence graph was constructed by plotting the maximum variance inflation factor (VIF) observed at each stepwise deletion round against the corresponding iteration number (Fig. 2 and Supplementary Table S2). For each round, the highest VIF among all retained predictors was extracted and used as a summary indicator of multicollinearity severity. A logarithmic scale was applied to the y-axis to accommodate the wide dynamic range of VIF values observed during early iterations (> 70,000) and to clearly visualize the monotonic decline and convergence toward acceptable levels (VIF ≤ 5) in the final model.

### Regression model choice

Out of the 115 animals contributing a total of 6868 semen quality records, statistical model convergence was achieved for 77 animals. Only these animals were retained for subsequent regression analyses and were therefore used in the canonical discriminant analysis (CDA) and CHAID classification analyses. Animals excluded at this stage were removed solely due to lack of model convergence and not because of missing phenotypic records, ensuring that downstream multivariate analyses were based on stable and reliable parameter estimates.

Supplementary Table S3 summarizes the comparative evaluation of the candidate regression models used to describe seminal quality traits. Models were assessed using a set of scale-harmonised criteria capturing goodness-of-fit, explanatory capacity, and predictive performance, including the percentage of successfully fitted curves (bucks for which model successfully converged), residual sum of squares (RSS), cross-validated minimum mean square prediction error (MMSE), and coefficients of determination (R^2^ and adjusted R^2^). In addition, the dispersion of adjusted R^2^ values across animals was examined to evaluate model stability at the individual buck level. Information-theoretic criteria (AIC, AICc, and BIC) were also computed to support model comparison while accounting for model complexity. Together, these complementary metrics provided a robust basis for identifying the regression model that best balanced fit quality, predictive accuracy, and consistency across bucks.

Unstandardized parameters were retained in the final regression models to preserve the natural biological scale of semen traits, enabling direct interpretation of values (e.g., milliliters for ejaculate volume or millions/mL for sperm concentration) and ensuring their practical application in breeding programs. This choice was supported by verification of key model assumptions, including normality, homoscedasticity, and independence of residuals, which together validate the cubic regression model as the most appropriate framework for describing the evolution of seminal quality traits in Murciano-Granadina bucks. Canonical discriminant analysis was conducted and interpreted separately, with results evaluated using eigenvalues, canonical correlations, and multivariate test statistics, without extending regression-based scaling considerations to the discriminant models.

The descriptive statistics presented in Table 1 provide a comprehensive overview of the distribution of parameters across traits, highlighting the broad variability observed in some measures such as sperm concentration (b_0_ ranging from − 66,335.71 to 92,233.72, mean 9223.37 ± 9223.37) and endosmosis (b_0_ from − 5994.72 to 2874.33, mean 5.24 ± 994.91), compared to more moderate variability in volume (b_0_ mean 2.35 ± 44.72) or Acrosome Integrity/Intact acrosomes (b_0_ mean 95.96 ± 145.09).

**Table 1:** Descriptive statistics for seminal quality curve unstandardized parameters derived from cubic regression analysis across Murciano-Granadina bucks.

	Mean	SD	Minimum	Maximum
Constant (b0) volume	2.35	44.72	− 128.17	269.16
b1 Volume	0.21	1.82	− 6.90	8.32
b2 Volume	0.00	0.02	− 0.13	0.04
b3 Volume	0.00	0.00	− 0.01	0.00
Constant (b0) sperm concentration	9223.37	9223.37	− 66,335.71	92,233.72
b1 sperm concentration	− 183.40	2601.08	− 9223.37	8420.98
b2 sperm concentration	6.19	83.71	− 318.66	284.05
b3 sperm concentration	− 0.32	3.35	− 11.09	14.04
Constant (b0) total motility	108.74	266.97	− 1175.34	1119.67
b1 total motility	− 3.15	17.58	− 96.53	24.25
b2 total motility	0.09	0.52	− 0.83	3.10
b3 total motility	0.00	0.01	− 0.02	0.04
Constant (b0) progressive motility	− 93.20	962.88	− 6391.74	654.93
b1 progressive motility	3.37	30.70	− 70.99	167.58
b2 progressive motility	− 0.01	0.33	− 1.12	0.88
b3 progressive motility	0.00	0.02	− 0.06	0.09
Constant (B0) acrosome integrity	95.96	145.09	− 414.12	856.42
b1 acrosome integrity	− 1.76	11.88	− 74.64	17.61
b2 acrosome integrity	0.05	0.17	− 0.22	0.71
b3 acrosome integrity	0.00	0.02	− 0.02	0.11
Constant (b0) endosmosis	5.24	994.91	− 5994.72	2874.33
b1 endosmosis	− 1.63	36.47	− 170.27	116.96
b2 endosmosis	0.00	1.11	− 5.23	2.53
b3 endosmosis	0.00	0.04	− 0.08	0.22
Constant (b0) total estimated volume	25.74	223.36	− 364.31	1475.50
b1 total estimated volume	0.26	7.90	− 38.17	34.34
b2 total estimated volume	0.01	0.10	− 0.30	0.33
b3 total estimated volume	0.00	0.01	− 0.04	0.00
Constant (b0) total doses	103.46	895.54	− 1457.84	5917.16
b1 total doses	1.03	31.65	− 153.09	137.42
b2 total doses	0.04	0.39	− 1.18	1.31
b3 total doses	− 0.01	0.03	− 0.17	0.01
Constant (b0) real added total volume	5.43	64.27	− 227.28	309.33
b1 real added total volume	0.73	3.97	− 7.34	21.42
b2 real added total volume	− 0.01	0.06	− 0.22	0.21
b3 real added total Volume	0.00	0.00	− 0.03	0.00
Constant (b0) real doses	21.20	256.58	− 908.12	1233.79
b1 real doses	2.97	15.86	− 29.29	85.59
b2 real doses	− 0.03	0.26	− 0.88	0.82
b3 real doses	0.00	0.02	− 0.11	0.02

In addition to the intercept (b_0_) and linear slope (b_1_), the quadratic (b_2_) and cubic (b_3_) terms were essential for capturing subtle curvature in the data, even when their mean values were close to zero. For volume, b_2_ ranged from − 0.13 to 0.04 (mean 0.00 ± 0.02) and b₃ from − 0.01 to 0.00 (mean 0.00 ± 0.00), indicating only minor departures from linearity. In contrast, sperm concentration showed more pronounced higher-order variability, with b_2_ spanning − 318.66 to 284.05 (mean 6.19 ± 83.71) and b₃ from − 11.09 to 14.04 (mean − 0.32 ± 3.35), reflecting non-linear fluctuations in this trait. Motility traits displayed modest curvature, with total motility b_2_ averaging 0.09 ± 0.52 and b_2_ at 0.00 ± 0.01, while progressive motility showed b_2_ at − 0.01 ± 0.33 and b_3_ at 0.00 ± 0.02. Endosmosis revealed stronger quadratic and cubic contributions (b_2_ mean 0.00 ± 1.11; b_3_ mean 0.00 ± 0.04), capturing the trait’s curvilinear variation. Dose-related parameters, including total doses (b_2_ mean 0.04 ± 0.39; b_3_ mean − 0.01 ± 0.03) and real doses (b_2_ mean − 0.03 ± 0.26; b_3_ mean 0.00 ± 0.02), followed the same trend of near-zero higher-order effects. Collectively, these results demonstrate that while the intercepts and linear slopes captured the main trajectory of change, the quadratic and cubic terms added precision by modeling subtle non-linearities, particularly in traits with greater biological variability such as sperm concentration and endosmosis. This confirms the cubic regression model with unstandardized parameters as the most reliable and biologically interpretable method for assessing seminal quality dynamics in Murciano-Granadina bucks.

### Data standardization and scaling strategy for multivariate analyses

Prior to multivariate analyses, the scaling strategy of explanatory variables was carefully evaluated. Canonical Discriminant Analysis (CDA) was performed using regression-derived coefficients (baseline ${b}_{0}$, linear ${b}_{1}$, quadratic ${b}_{2}$, and cubic ${b}_{3}$) obtained from cubic regression models fitted separately for each seminal quality trait. These coefficients, rather than raw semen measurements, were used as explanatory variables to characterize the dynamic behavior of seminal parameters over time, following approaches recommended for longitudinal or trajectory-based phenotypes^25,26^.

Because all variables entered into the CDA represented homogeneous regression parameters within each coefficient order, and not original phenotypic traits with heterogeneous units, no external standardization (e.g., z-score normalization) was applied prior to analysis. Retaining unstandardized coefficients allowed preservation of their biological scale and interpretability, facilitating direct inference in terms of semen volume (mL), sperm concentration (×10^6^ spermatozoa/mL), motility (%), and membrane functionality (%), which is particularly relevant for reproductive management and breeding applications^27,28^.

Within the CDA framework, standardized canonical coefficients and structure loadings were computed internally, ensuring scale-free comparison of variable contributions to group discrimination^29^. In addition, multicollinearity among predictors was rigorously controlled through an iterative Variance Inflation Factor (VIF)–based elimination procedure, resulting in a final predictor set with acceptable tolerance values (VIF ≤ 5), consistent with widely accepted thresholds in multivariate modeling^30,31^. This approach minimized redundancy and reduced the risk of scale-related dominance effects.

Overall, this strategy ensured that discrimination among αS1- and κ-casein genotypes was driven by biologically meaningful variation in seminal quality dynamics rather than by artefacts of variable scaling, while maintaining statistical robustness and interpretability.

### Canonical correlation dimensions, efficiency and model reliability

#### Canonical correlation dimensions

For the αS1-casein genotype, four canonical functions were identified. The first canonical function (F1) showed the highest canonical correlation (*r* = 0.675), indicating a strong relationship between the discriminant scores and genotype classification. The second function (F2) also exhibited a substantial canonical correlation (*r* = 0.599), followed by progressively lower correlations for the third (F3: *r* = 0.472) and fourth (F4: *r* = 0.270) functions. This pattern reflects a gradual decline in discriminatory strength across successive canonical dimensions.

For the κ-casein genotype, two canonical functions were obtained. The first function (F1) showed a canonical correlation of 0.554, while the second function (F2) exhibited a slightly lower correlation (0.492), indicating moderate discriminatory power for both axes, with stronger separation along the first canonical dimension.

**Fig. 3:**
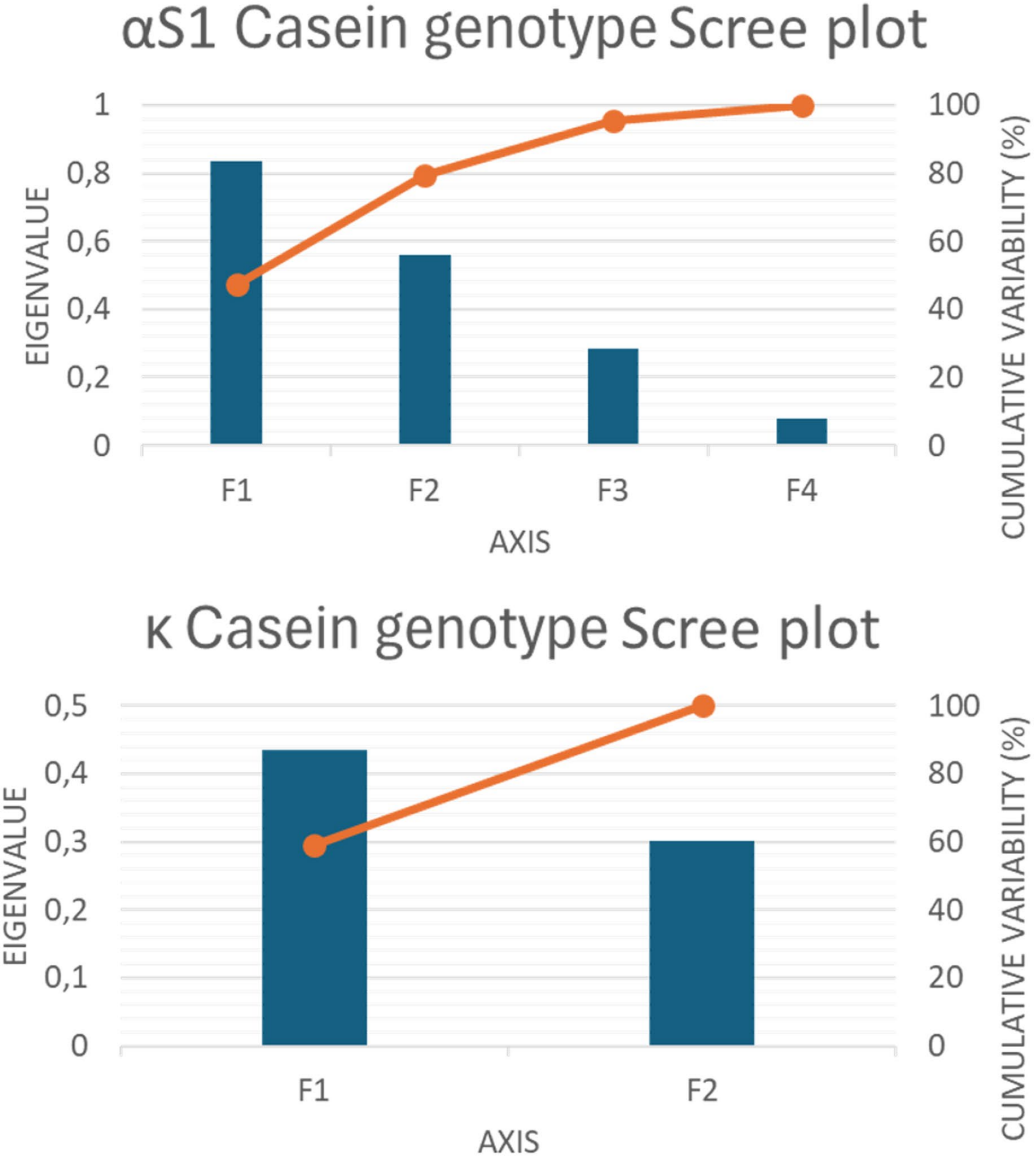
Canonical variable function eigenvalues and cumulative variability across functions/dimensions identified by discriminant canonical analysis.

Overall, the canonical correlation structure supports the interpretation that most of the discriminative information is concentrated in the first one or two canonical functions for both genotypes, with diminishing contributions from subsequent functions. This pattern is visually confirmed by the scree plots (Fig. 3), which display the eigenvalues (bars) of the first four canonical functions (F1–F4) for the α.

The scree plots for αS1-casein and κ-casein genotypes (Fig. 3) reveal clear differences in the distribution of explained variability across principal axes. For αS1-casein genotype, the first axis (F1) has an eigenvalue of 0.835, accounting for 47.463% of the total discrimination. The second axis (F2) shows an eigenvalue of 0.559, explaining an additional 31.784%, bringing the cumulative discrimination to 79.247%. The third axis (F3) contributes an eigenvalue of 0.286 (16.270%), while the fourth axis (F4) has a much smaller eigenvalue (0.079, 4.483%), with cumulative discrimination reaching 100%. These results indicate that the first two axes capture the majority of the meaningful structure in αS1-casein genotype variability.

In contrast, the κ-casein genotype scree plot displays a simpler two-axis structure. The first axis (F1) has an eigenvalue of 0.435, explaining 59.022% of the total discrimination, while the second axis (F2) has an eigenvalue of 0.302, accounting for the remaining 40.978%, together explaining 100% of the total variability. The marked dominance of the first axis indicates that most of the discriminative information for κ-casein genotypes is concentrated along F1.

### CDA model adequacy and reliability

#### Bartlett’s test

In the present analysis, Bartlett’s tests were significant for all retained canonical functions, confirming the statistical validity of the discriminant structure for both genotypes (Table 2). For the αS1-casein genotype, the first canonical function (F1) showed the highest eigenvalue (0.835) and was highly significant (Bartlett’s statistic = 42.050, *p* < 0.01), followed by the second function (F2), which also exhibited a substantial eigenvalue (0.559) and remained statistically significant (Bartlett’s statistic = 23.537, *p* < 0.01). The third (F3; eigenvalue = 0.286, Bartlett’s statistic = 9.992, *p* < 0.01) and fourth (F4; eigenvalue = 0.079, Bartlett’s statistic = 2.315, *p* < 0.01) canonical functions were likewise statistically significant, but their markedly lower eigenvalues indicate a progressively reduced contribution to the overall discriminatory structure.

**Table 2:** Canonical discriminant analysis efficiency parameters showing the significance of each canonical discriminant function based on Bartlett’s test.

Genotype	Parameter	F1	F2	F3	F4
αS1 Casein	Eigenvalue	0.835	0.559	0.286	0.079
	Bartlett’s statistic	42.050	23.537	9.992	2.315
κ Casein	Eigenvalue	0.435	0.302		
	Bartlett’s statistic	20.638	8.713		

All results were statitically significant (*P* < 0.01).

For the κ-casein genotype, Bartlett’s tests also supported the significance of both canonical functions. The first function (F1) dominated the discriminant structure (eigenvalue = 0.435; Bartlett’s statistic = 20.638, *p* < 0.01), while the second function (F2) showed a smaller but still statistically significant contribution (eigenvalue = 0.302; Bartlett’s statistic = 8.713, *p* < 0.01).

Overall, although Bartlett’s tests confirm that multiple canonical functions are statistically significant, practical interpretation focuses primarily on the first one or two functions, where statistical significance coincides with higher eigenvalues and greater discriminatory power.

#### Wilks’ lambda test

The Wilks’ Lambda analysis demonstrates that αS1 and κ casein genotypes have meaningful effects on seminal quality, with several regression coefficients showing statistical significance. For αS1 casein, the b0 (Constant) coefficient for Total Real Added Volume (*p* = 0.023) reflects a significant effect of the genotype on the baseline semen volume, suggesting that males carrying αS1 may produce greater ejaculate volume. Similarly, the b0 coefficient for Progressive Motility (*p* = 0.043) indicates that αS1 genotype positively influences baseline sperm motility, a key determinant of fertilization capacity. Trends in b2 coefficients for Progressive Motility (*p* = 0.079) and Constant Endosmosis (*p* = 0.099) suggest that αS1 may also affect the rate of change or curvature in motility and sperm membrane functionality over the measured period, although these effects are less pronounced.

For κ casein, significant effects were observed for b2 Volume (*p* = 0.045) and b0 Total Real Added Volume (*p* = 0.050). The b0 effect again indicates an influence on baseline semen volume, while the b2 effect reflects a potential role in modulating the rate of change in semen volume, which could be important for ejaculate consistency or production dynamics.

Overall, these findings indicate that αS1-casein genotype is associated with limited but consistent differences in semen volume and sperm motility, while κ-casein genotype shows a more restricted association primarily with semen volume. The significant regression coefficients identified for specific traits reflect differences in baseline levels as well as in selected dynamic components of seminal quality, rather than broad effects across all parameters. Taken together, these results suggest that casein genotypes are linked to trait-specific patterns in seminal quality dynamics, supporting their biological relevance in male reproductive performance without implying direct or causal effects (Table 3).

**Table 3:** Results for the tests of equality of group means to test for difference in the means across αS1- and κ-Casein genotypes once redundant variables have been removed.

Genotype	Variable	Wilks’ Lambda	F	DF1	DF2	*p*-value	Rank
αS1 Casein	Constant real total added volume	0.723	3.252	4	34	0.023	1
	Constant progressive motility	0.755	2.763	4	34	0.043	2
	b2 progressive motility	0.788	2.293	4	34	0.079	3
	Constant endosmosis	0.800	2.126	4	34	0.099	4
	b2 total estimated volume	0.843	1.589	4	34	0.200	5
	Constant sperm concentration	0.861	1.373	4	34	0.264	6
	b3 endosmosis	0.904	0.900	4	34	0.475	7
	Constant acrosome integrity	0.933	0.606	4	34	0.661	8
	b2 volume	0.942	0.522	4	34	0.720	9
	b2 acrosome integrity	0.954	0.412	4	34	0.799	10
κ Casein	b2 volume	0.862	2.971	2	37	0.045	1
	Constant total real added volume	0.869	2.782	2	37	0.050	2
	b2 abnormal sperm cpunt	0.891	2.252	2	37	0.119	3
	Constant sperm concentration	0.902	2.011	2	37	0.148	4
	b2 progressive motility	0.947	1.028	2	37	0.368	5
	Constant endosmosis	0.956	0.843	2	37	0.439	6
	b3 endosmosis	0.961	0.760	2	37	0.475	7
	b2 total estimated volume	0.978	0.418	2	37	0.661	8
	Constant acrosome integrity	0.993	0.124	2	37	0.884	9

#### Pillai’s trace criterion and false discovery rate (FDR)

The multivariate analysis based on Pillai’s Trace criterion indicated a statistically significant but limited overall effect of both αS1-casein and κ-casein genotypes on the set of seminal quality regression coefficients (Table 4). For αS1-casein, Pillai’s Trace was 0.549 with an observed F value of 1.096 (*p* = 0.049), while for κ-casein, Pillai’s Trace was 0.535 with an observed F of 1.218 (*p* = 0.048). Although statistically significant at the 5% level, these results indicate that casein genotypes explain only a limited proportion of the joint multivariate variation in the cubic regression coefficients describing seminal traits.

**Table 4:** Multivariate test statistics (Pillai’s Trace criterion) for αS1 and κ casein genotypes, showing significant differences across semen quality traits at α = 0.05.

Genotype	αS1 Casein	κ Casein
Pillai’s trace criterion	0.549	0.535
F (observed value)	1.096	1.218
F (critical value)	1.754	1.778
DF1	20	18
DF2	58	60
p-value	0.049	0.048

Importantly, the presence of a limited global multivariate effect does not preclude more pronounced genotype-specific influences on individual regression coefficients. Accordingly, univariate Wilks’ Lambda analyses were used to identify specific seminal traits whose coefficients contributed to group separation across αS1- and κ-casein genotypes. Table 3 reports the Wilks’ Lambda statistics for the retained explanatory variables, providing effect-size-oriented information on the contribution of individual coefficients to genotype discrimination. To address concerns related to multiple testing when interpreting these univariate effects, Supplementary Table S4 presents the corresponding false discovery rate (FDR) adjustment of associated p-values using the Benjamini–Hochberg procedure. After FDR correction, the majority of the retained variables remained statistically significant, indicating that the univariate effects reported in Table 3 are not driven by chance or isolated p-values. Taken together, these results show that, although the overall multivariate genotype effect is limited, several individual regression coefficients exhibit robust genotype-associated differences supported both by effect-size measures (Wilks’ Lambda) and by significance that persists after correction for multiple comparisons.

#### Canonical standardized coefficients, loadings, and spatial representation

The standardized discriminant coefficients in Table 5 indicate that αS1 and κ casein genotypes differentially influence seminal quality traits, with clear biological implications. For αS1 casein, the first canonical function (F1) is most strongly influenced by Constant Sperm Concentration (0.610), Constant Progressive Motility (0.333), and Constant Real Total Added Volume (0.316), highlighting its role in enhancing baseline ejaculate output and fertilizing potential. Negative contributions from b2 Acrosome Integrity/Intact acrosomes (− 0.623) and b2 Volume (0.390) suggest that αS1 also modulates sperm quality and the dynamics of semen volume over time, while the second canonical function (F2) is dominated by b3 Endosmosis (0.725), reflecting effects on sperm membrane integrity and osmotic tolerance. For κ casein, F1 is primarily shaped by Constant Sperm Concentration (0.591), Constant Acrosome Integrity/Intact acrosomes (0.373), and b3 Endosmosis (0.242), whereas F2 is driven by b3 Endosmosis (0.807) and Constant Total Real Added Volume (0.592), indicating a strong influence on sperm membrane functionality and semen volume. Overall, these results demonstrate that αS1 casein mainly supports baseline semen volume and motility, while κ casein enhances sperm membrane stability and ejaculate volume, highlighting the distinct biological roles of casein genotypes in male fertility.

**Table 5:** Standardized canonical discriminant coefficients (β) for the first (F1) and second (F2) functions by αS1- and κ-casein genotypes, indicating the relative contribution and biological relevance of semen traits in genotype discrimination.

Casein genotype	Variable	F1 standardized coefficient (β)	F2 standardized coefficient (β)	Biological interpretation
αS1-Casein	b2 volume	0.390	**− 0.910**	F1: Higher volume favors this genotype; F2: higher volume reduces the secondary discriminant score
	Constant sperm concentration	**0.610**	**− 0.521**	F1: Higher concentration characteristic of this genotype; F2: higher concentration slightly reduces F2 score
	b2 progressive motility	− 0.007	− 0.264	Minimal effect on F1; negative on F2 → higher motility slightly reduces F2 score
	Constant acrosome integrity	0.269	**0.494**	Positive on both → higher Acrosome Integrity contributes to discriminating this genotype (less typical biologically, but statistically contributes)
	b2 acrosome integrity	**− 0.623**	− 0.358	Strong negative → higher Acrosome Integrity reduces the discriminant score → lower sperm defects favor genotype
	Constant endosmosis	− 0.189	− 0.185	Slight negative → lower endosmosis (membrane stability) slightly reduces scores
	b3 endosmosis	0.232	**0.725**	Positive → better sperm viability/endosmosis strongly contributes to F2
	b2 total estimated volume	0.079	0.282	Small positive effect → higher total volume slightly favors genotype
	Constant real total added volume	0.316	**0.473**	Positive → larger processed/usable volume favors genotype
	Constant progressive motility	0.333	**− 0.544**	F1: higher motility favors genotype; F2: higher motility reduces secondary score
	Variable	F1 Standardized Coefficient (β)	F2 Standardized Coefficient (β)	Biological Interpretation
κ-Casein	b2 volume	0.297	**− 0.915**	F1: higher volume slightly favors genotype; F2: higher volume reduces secondary discriminant score
	Constant sperm concentration	**0.591**	**− 0.629**	Positive F1 → higher concentration characteristic; negative F2 → reduces secondary score
	b2 progressive motility	− 0.031	− 0.262	Small negative → higher motility slightly reduces discriminant scores
	Constant acrosome integrity	0.373	0.344	Positive → higher Acrosome Integrity contributes to separating genotype
	b2 acrosome integrity	**− 0.706**	− 0.193	Strong negative → lower Acrosome Integrity favors genotype
	Constant endosmosis	− 0.006	**− 0.566**	Minimal F1 effect; negative F2 → lower membrane stability reduces secondary score
	b3 endosmosis	0.242	**0.807**	Positive → better sperm viability strongly favors genotype in F2
	b2 total estimated volume	0.129	0.223	Small positive → higher total volume slightly favors genotype.
	Constant total real added volume	0.282	**0.592**	Positive → larger usable volume favors genotype
	Constant progressive motility	0	0	Not relevant for in κ Casein analysis

Bold: All variables were included in the estimation of the canonical discriminant functions; however, interpretation focused on variables with substantive structure loadings and variables with absolute structure loadings ≥ |0.40|, consistent with common practice.

These standardized coefficients were used to build discriminant functions for both genotypes as follows;

αS1 Casein genotype

$$\begin{array}{l}{\rm{F1 }} = {\rm{ }}0.{\rm{39}}0{\rm{ }}*{\rm{ b2 Volume}}\\\quad \quad + {\rm{ }}0.{\rm{61}}0{\rm{ }}*{\rm{ Constant Sperm Concentration}}\\\quad \quad - {\rm{ }}0.00{\rm{7 }}*{\rm{ b2 Progressive Motility}}\\\quad \quad + {\rm{ }}0.{\rm{269 }}*{\rm{ Constant Acrosome Integrity}}/{\rm{Intact acrosomes}}\\\quad \quad - {\rm{ }}0.{\rm{623 }}*{\rm{ b2 Acrosome Integrity}}/{\rm{Intact acrosomes}}\\\quad \quad - {\rm{ }}0.{\rm{189 }}*{\rm{ Constant Endosmosis}}\\\quad \quad + {\rm{ }}0.{\rm{232 }}*{\rm{ b3 Endosmosis}}\\\quad \quad + {\rm{ }}0.0{\rm{79 }}*{\rm{ b2 Total Estimated Volume}}\\\quad \quad + {\rm{ }}0.{\rm{316 }}*{\rm{ Constant Real Total Added Volume}}\\\quad \quad + {\rm{ }}0.{\rm{333 }}*{\rm{ Constant Progressive Motility}}\end{array}$$

$$\begin{array}{l}{\rm{F2 }} = {\rm{ }} - 0.{\rm{91}}0{\rm{ }}*{\rm{ b2 Volume}}\\\quad \quad - {\rm{ }}0.{\rm{521 }}*{\rm{ Constant Sperm Concentration}}\\\quad \quad - {\rm{ }}0.{\rm{264 }}*{\rm{ b2 Progressive Motility}}\\\quad \quad + {\rm{ }}0.{\rm{494 }}*{\rm{ Constant Acrosome Integrity}}/{\rm{Intact acrosomes}}\\\quad \quad - {\rm{ }}0.{\rm{358 }}*{\rm{ b2 Acrosome Integrity}}/{\rm{Intact acrosomes}}\\\quad \quad - {\rm{ }}0.{\rm{185 }}*{\rm{ Constant Endosmosis}}\\\quad \quad + {\rm{ }}0.{\rm{725 }}*{\rm{ b3 Endosmosis}}\\\quad \quad + {\rm{ }}0.{\rm{282 }}*{\rm{ b2 Total Estimated Volume}}\\\quad \quad + {\rm{ }}0.{\rm{473 }}*{\rm{ Constant Real Total Added Volume}}\\\quad \quad - {\rm{ }}0.{\rm{544 }}*{\rm{ Constant Progressive Motility}}\end{array}$$

$$\begin{array}{l}{\rm{F1 }} = {\rm{ }}0.{\rm{297 }}*{\rm{ b2 Volume}}\\\quad \quad + {\rm{ }}0.{\rm{591 }}*{\rm{ Constant Sperm Concentration}}\\\quad \quad - {\rm{ }}0.0{\rm{31 }}*{\rm{ b2 Progressive Motility}}\\\quad \quad + {\rm{ }}0.{\rm{373 }}*{\rm{ Constant Acrosome Integrity}}/{\rm{Intact acrosomes}}\\\quad \quad - {\rm{ }}0.{\rm{7}}0{\rm{6 }}*{\rm{ b2 Acrosome Integrity}}/{\rm{Intact acrosomes}}\\\quad \quad - {\rm{ }}0.00{\rm{6 }}*{\rm{ Constant Endosmosis}}\\\quad \quad + {\rm{ }}0.{\rm{242 }}*{\rm{ b3 Endosmosis}}\\\quad \quad + {\rm{ }}0.{\rm{129 }}*{\rm{ b2 Total Estimated Volume}}\\\quad \quad + {\rm{ }}0.{\rm{282 }}*{\rm{ Constant Total Real Added Volume}}\end{array}$$

$$\begin{array}{l}{\rm{F2 }} = {\rm{ }} - 0.{\rm{915 }}*{\rm{ b2 Volume}}\\\quad \quad - {\rm{ }}0.{\rm{629 }}*{\rm{ Constant Sperm Concentration}}\\\quad \quad - {\rm{ }}0.{\rm{262 }}*{\rm{ b2 Progressive Motility}}\\\quad \quad + {\rm{ }}0.{\rm{344 }}*{\rm{ Constant Acrosome Integrity}}/{\rm{Intact acrosomes}}\\\quad \quad - {\rm{ }}0.{\rm{193 }}*{\rm{ b2 Acrosome Integrity}}/{\rm{Intact acrosomes}}\\\quad \quad - {\rm{ }}0.{\rm{566 }}*{\rm{ Constant Endosmosis}}\\\quad \quad + {\rm{ }}0.{\rm{8}}0{\rm{7 }}*{\rm{ b3 Endosmosis}}\\\quad \quad + {\rm{ }}0.{\rm{223 }}*{\rm{ b2 Total Estimated Volume}}\\\quad \quad + {\rm{ }}0.{\rm{592 }}*{\rm{ Constant Total Real Added Volume}}\end{array}$$

**Fig. 4:**
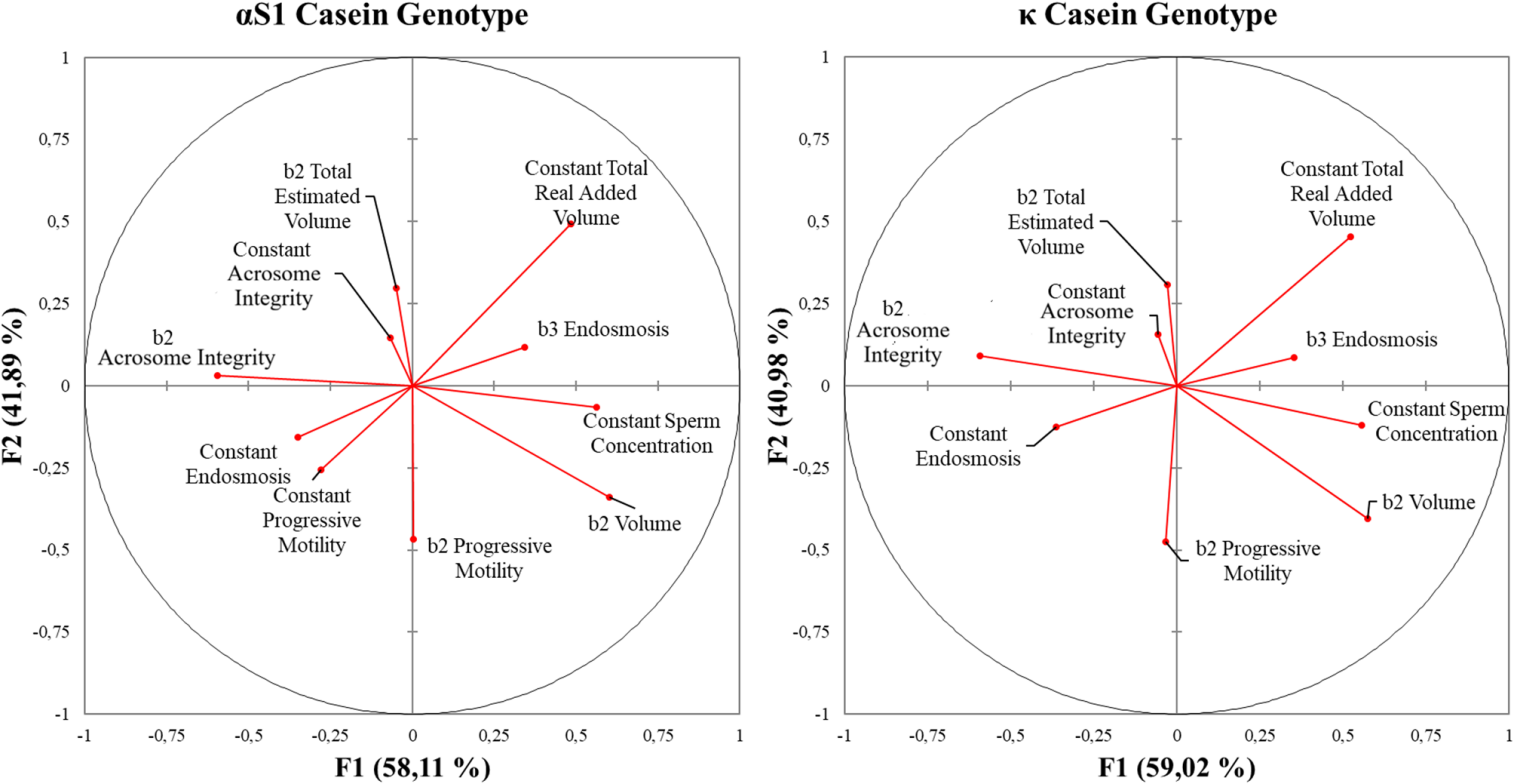
Vector plots for discriminant loadings for semen quality-related traits across αS1- (left) and κ- (right) Casein genotypes.

Discriminant loading vectors for semen quality traits across αS1- (left) and κ- (right) casein genotypes. For αS1-casein, Function 1 accounts for 64.2% and Function 2 for 35.8% of the variability, indicating that discrimination is strongly driven by the first axis. The longest vectors correspond to traits with loadings above 0.6 on Function 1, highlighting their major role in genotype separation, while traits with negative loadings around − 0.4 to − 0.5 contribute in the opposite direction. The wider angular dispersion of vectors suggests higher heterogeneity in how traits influence differentiation. In contrast, for κ-casein, Function 1 explains 58.4% and Function 2 41.6% of the variability. Vector magnitudes are generally lower, with most clustering between ± 0.3 and ± 0.6, reflecting weaker but more evenly distributed discriminant power. The narrower angular spread implies more consistent contributions across traits. Overall, αS1-casein genotypes are more distinctly and heterogeneously discriminated by semen quality traits than κ-casein genotypes, where differentiation is more balanced but weaker (Fig. 4).

**Fig. 5:**
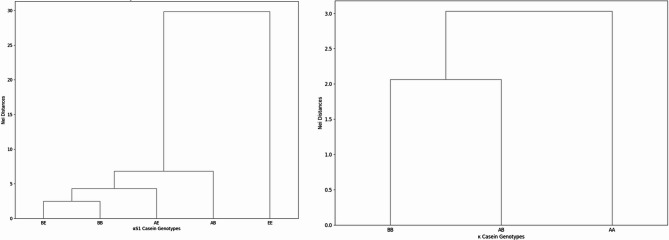
Dendrogram constructed from Mahalanobis’s distances across αS1- (left) and κ- (right) Casein genotypes.

Figure 5 displays the hierarchical clustering of αS1- (left) and κ- (right) casein genotypes based on Nei’s genetic distances. In αS1-casein, genotypes show a broad separation, with EE forming the most distinct cluster, while BE and BB group closely together, followed by AE and AB at intermediate distances. The large distance values (up to ~ 30) indicate considerable genetic differentiation among αS1-casein genotypes. In contrast, κ-casein genotypes exhibit much shorter distances (maximum ~ 3), reflecting closer genetic relationships. Here, BB and AB cluster first, while AA remains more distinct but still within a narrow divergence range. These patterns suggest that αS1-casein genotypes display higher genetic variability and stronger structuring than κ-casein genotypes, which appear comparatively more homogeneous.

### CDA function leave-one-out cross-validation

Canonical discriminant analysis (CDA) evaluated by leave-one-out cross-validation (LOO-CV) revealed marked differences in classification performance between αs1-casein and κ-casein genotypes as shown in confusion matrices in Supplementary Table 5. For αs1-casein, overall classification accuracy was high (74.03%), driven largely by the dominant BE genotype, which was classified with 100% accuracy. In contrast, minority genotypes (AB, AE, BB, EE, and AA) exhibited substantial misclassification and were predominantly assigned to the BE class, reflecting pronounced class imbalance and limited discriminative separation among rare genotypes. For κ-casein, CDA achieved a moderate overall accuracy (64.94%), with the AB genotype showing high correct classification (94.6%), whereas AA and BB genotypes displayed considerable overlap and lower classification success.

Chance-corrected evaluation using Press’s Q statistic indicated that classification performance exceeded random expectation for both loci. Press’s Q values exceeded the chi-square critical threshold (χ^2^ = 6.63, df = 1, *p* < 0.01), corresponding to at least 25% improvement over chance classification. Specifically, classification accuracy was significantly greater than random for αs1-casein (Q = 182.40, df = 5, *p* < 0.05) and for κ-casein (Q = 34.60, df = 2, *p* < 0.05), confirming statistically meaningful—though uneven—discriminatory performance, with markedly stronger separation observed for αs1-casein genotypes.

### Effect size-based discriminant contributions of casein genotypes

Effect size estimates derived from univariate discriminant tests revealed heterogeneous contributions of semen quality regression components to genotype discrimination for αS1- and κ-casein genotypes (Table 6). Global multivariate tests (Wilks’ Lambda, Pillai’s trace, Hotelling–Lawley trace) were not statistically significant in either analysis, indicating limited overall separation among genotype groups. Accordingly, interpretation emphasized component-specific effect sizes and confidence intervals rather than global discrimination statistics.

**Table 6:** Effect sizes, 95% confidence intervals, and interpretation for discriminant contributions of semen-trait regression components for αS1and κ-casein genotypes.

Casein genotype	Variable	F	df1	df2	*p*-value	ηp²	95% CI for ηp^2^	Effect size interpretation
αS1- Casein	b2 volume	0.522	4	34	0.720	0.058	0.00–0.20	Small
	Constant sperm concentration	1.373	4	34	0.264	0.139	0.00–0.31	Moderate
	b2 progressive motility	2.763	4	34	0.043	0.245	0.02–0.45	Moderate–large
	Constant acrosome integrity	2.293	4	34	0.080	0.212	0.00–0.41	Moderate–large
	b2 acrosome integrity	0.606	4	34	0.661	0.067	0.00–0.23	Moderate
	Constant endosmosis	0.412	4	34	0.799	0.046	0.00–0.18	Small
	b3 endosmosis	2.126	4	34	0.099	0.200	0.00–0.39	Moderate–large
	b2 total estimated volume	0.901	4	34	0.475	0.096	0.00–0.27	Moderate
	Constant real total added volume	1.589	4	34	0.200	0.158	0.00–0.34	Moderate–large
	Constant progressive motility	3.252	4	34	0.023	0.277	0.03–0.48	Large
κ- Casein	b2 volume	2.971	2	37	0.064	0.138	0.00–0.33	Moderate
	Constant sperm concentration	2.782	2	37	0.075	0.131	0.00–0.32	Moderate
	b2 progressive motility	2.252	2	37	0.119	0.108	0.00–0.28	Moderate
	Constant acrosome integrity	2.011	2	37	0.148	0.098	0.00–0.26	Moderate
	b2 acrosome integrity	1.028	2	37	0.368	0.053	0.00–0.18	Small
	Constant endosmosis	0.843	2	37	0.439	0.044	0.00–0.16	Small
	b3 endosmosis	0.760	2	37	0.475	0.039	0.00–0.15	Small
	b2 total estimated volume	0.418	2	37	0.661	0.022	0.00–0.12	Small
	Constant total real added volume	0.124	2	37	0.884	0.007	0.00–0.06	Negligible

For αS1-casein, baseline components related to progressive motility and total recoverable semen volume exhibited moderate to large effect sizes, with ηp^2^ values indicating that these traits accounted for a substantial proportion of between-genotype variability despite overlapping confidence intervals. Several dynamic components, including curvature terms for motility and total estimated volume, showed moderate effect sizes, suggesting genotype-dependent differences in age-related semen quality trajectories. Other components displayed small or statistically uncertain effects.

In contrast, κ-casein genotypes were characterized predominantly by small to moderate effect sizes, particularly for volume-related dynamic components and baseline recoverable volume. Motility, membrane integrity, and sperm abnormality components showed consistently small ηp^2^ values with wide confidence intervals, indicating limited discriminatory contribution in this dataset.

The combined analysis (Table 6) demonstrates that effect size-based interpretation provides biologically informative contrasts between casein genotype, with αS1-casein showing stronger associations with both baseline semen quality and dynamic aging patterns than κ-casein. These findings support the use of regression-derived trait components and effect size estimation as complementary tools for exploring genotype–phenotype relationships in reproductive traits.

### CHAID decision tree data mining

**Fig. 6:**
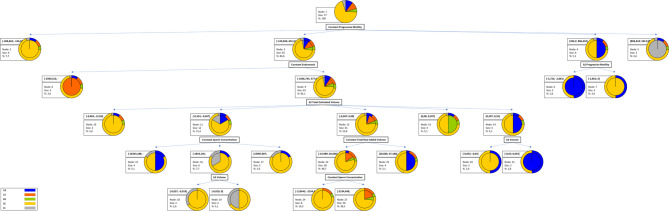
CHAID decision tree for seminal quality regression curve parameters across αS1 Casein genotype possibilities.

**Fig. 7:**
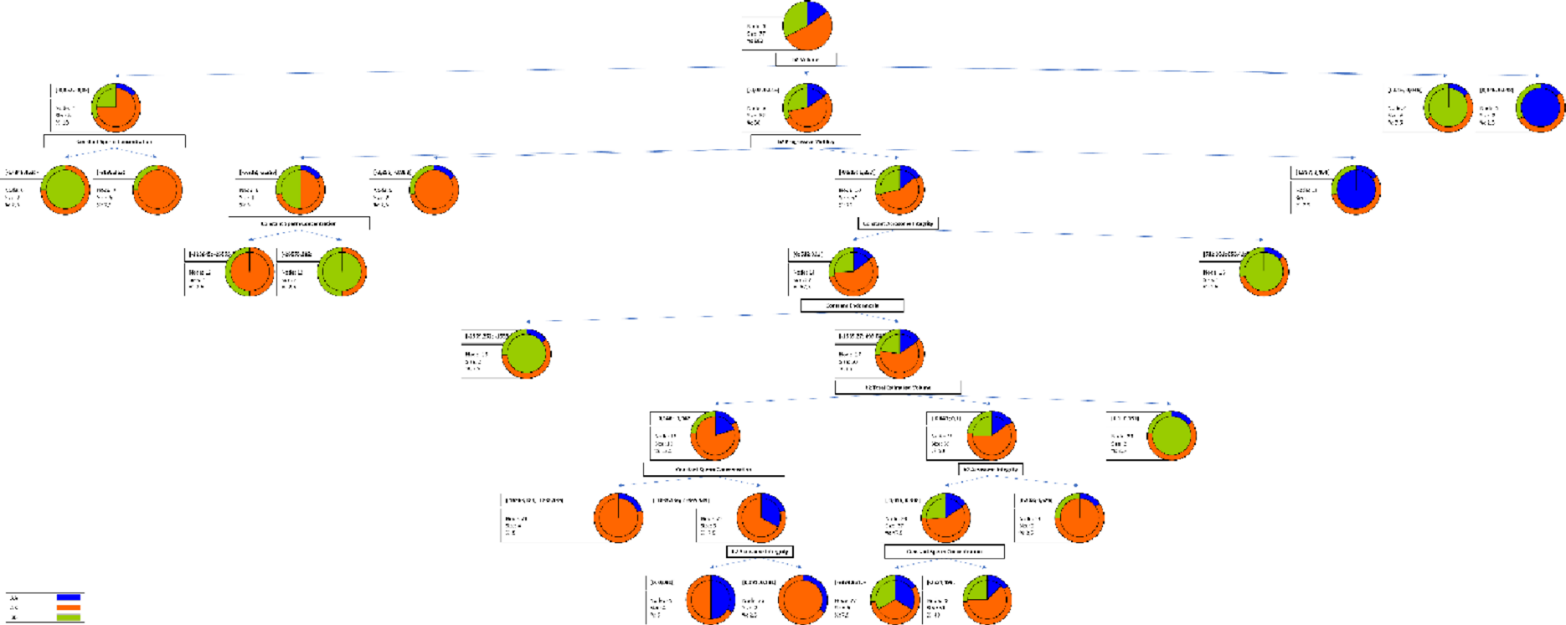
CHAID decision tree for seminal quality regression curve parameters across κ Casein genotype possibilities.

The decision tree analyses revealed distinct patterns in seminal quality traits associated with αs1-casein and κ-casein genotypes in Murciano-Granadina bucks (Figs. 6 and 7).

αs1-Casein Genotypes (Fig. 6).

The distribution of seminal traits across αs1-casein genotypes (Fig. 6) showed marked differences in progressive motility, sperm concentration, and ejaculate volume.

- Genotype BE was characterized by high variability in progressive motility and comparatively low baseline sperm concentration, as reflected by lower intercept coefficients relative to other genotypes, together with regression coefficients indicating potential declines in ejaculate volume (*b*_2_ Ejaculate Volume). Taken together, these coefficient patterns indicate a lower predicted semen-quality profile, quantified by reduced baseline levels and less favorable dynamic terms for key traits such as motility, concentration, and volume. Notably, the substantial within-genotype variability observed for motility suggests that individual BE males may still exhibit favorable semen characteristics despite the overall pattern.
- Genotype EE exhibited the highest baseline Progressive Motility and potential for elevated sperm concentration, but decision paths also indicated a downward concavity in total estimated volume (b2 Total Estimated Volume), suggesting long-term decreases.
- Genotype AB showed higher baseline total real added volume, although associated with low Sperm Concentration and possible declines in Progressive Motility over time.
- Genotype AE displayed a more favorable baseline Sperm Concentration compared to AB and BE, with potential increases in ejaculate volume (b2 Ejaculate Volume). Variability across nodes, however, suggests the need for careful management.
- Genotype BB presented a profile similar to AE, with moderate Progressive Motility, better baseline Sperm Concentration, and potential for increased ejaculate volume.

Taken together, EE and AB emerged as the most valuable αs1-casein genotypes for breeding purposes, while AE and BB were moderately valuable, and BE appeared the least advantageous.

κ-Casein Genotypes (Fig. 7).

The κ-casein genotype tree (Fig. 7) highlighted complementary associations between ejaculate volume, sperm concentration, and structural integrity traits.

- Genotype AA showed high Sperm Concentration, stable Progressive Motility, and consistently better acrosome integrity (% intact acrosomes) and endosmosis, despite a moderate ejaculate volume. These findings underline its role as a high-quality semen genotype.
- Genotype AB represented an intermediate profile, with balanced fertility potential, good ejaculate volume, and stable Progressive Motility, but higher variability in Sperm Concentration. This makes AB a versatile option for general-purpose breeding.
- Genotype BB was associated with the highest ejaculate volume, providing advantages for semen collection and large-scale AI programs. However, this was offset by lower Progressive Motility, Sperm Concentration, and Acrosome Integrity, indicating reduced fertilization efficiency without strict quality control.

### αS1 and κ-Casein genotypes integrated assessment

When considered jointly, αS1–κ-casein genotype combinations showed heterogeneous associations with semen quality traits; however, these patterns should be interpreted cautiously given the limited sample sizes for several combinations. Rare combinations such as EE–AA (*n* = 1) and AE–AA (*n* = 1) were associated with high values for sperm motility, concentration, and structural integrity, but the very small number of observations precludes any generalization beyond descriptive reporting. Similarly, AB–AB (*n* = 3) and BB–AB (*n* = 3) combinations were characterized by more balanced profiles, with stable ejaculate volumes and intermediate quality parameters, although uncertainty remains high due to low replication. The BB–BB combination (*n* = 3) was associated with higher semen volume accompanied by comparatively lower sperm quality traits, suggesting a potential trade-off between yield and quality under these genotypic backgrounds. More frequent combinations, such as BE–AB (*n* = 26), displayed consistent intermediate-to-high semen quality across traits, while EE–AB (*n* = 1) again represents an isolated observation. Overall, these results describe genotype-combination–specific patterns in semen quality parameters within the studied population, but they do not constitute evidence of superiority for artificial insemination or extensive breeding systems in the absence of fertility outcomes, temporal stability analyses, or external validation.

### CHAID accuracy and ten-fold cross-validation

CHAID classification trees evaluated under 10-fold cross-validation revealed genotype-specific differences in predictive performance for κ-casein and αs1-casein (Figs. 6 and 7). Using Press’s Q statistic to assess chance-corrected accuracy, the κ-casein tree yielded Q = 19.64 (df = 2) and the αs1-casein tree yielded Q = 143.44 (df = 4), both exceeding the corresponding chi-square critical values at α = 0.05, indicating classification performance significantly better than random expectation and markedly stronger discrimination for αs1-casein.

At the genotype level, the αs1-casein CHAID model showed its highest classification accuracy for the dominant BE genotype (89.80%), while minority genotypes (AB, AE, and EE) exhibited lower but non-negligible correct classification rates (≈ 14–30%). Notably, the rare EE genotype showed comparatively higher classification success (66.67%), although this result should be interpreted cautiously due to its limited sample size. In contrast, the κ-casein CHAID model demonstrated moderate and more evenly distributed discrimination, with AB and BB genotypes showing intermediate correct classification rates (65.69% and 54.84%, respectively), while the AA genotype exhibited the lowest classification success (25%), reflecting persistent overlap among κ-casein classes.

Consistent with these genotype-level patterns, the κ-casein model showed a higher misclassification risk (0.429; SE = 0.079, accuracy 57.14%) than the αs1-casein model (0.325; SE = 0.074, accuracy 67.53%). Identical risk estimates under resubstitution and cross-validation indicated stable tree structures without evidence of overfitting. Overall, these findings confirm that the explanatory variables provide greater discriminatory information for αs1-casein, particularly for the BE genotype, whereas κ-casein genotypes exhibit broader overlap and reduced separability.

## Discussion

Caseins are the predominant proteins in milk, providing essential amino acids and playing a central role in nutrition^32^. Their industrial significance, particularly in cheese production^21^, has made them a key target in the Murciano-Granadina genetic breeding programme^33^. Within this framework, males are routinely genotyped for caseins to ensure favorable alleles are transmitted through artificial insemination^15,34^.

While selection in this breed has historically prioritized morphology and genetic values for milk production^35,36^, semen quality has received comparatively less attention. Given the high economic and genetic value of sires, the identification of reliable predictors of semen quality—particularly genetic markers such as casein variants—would be highly advantageous, allowing improved reproductive efficiency and a more rational use of artificial insemination resources. However, exploiting such predictors requires statistical models capable of separating biologically meaningful variation from redundancy inherent to highly correlated seminal traits. In this context, careful control of multicollinearity was essential to ensure that inferred relationships reflected true biological structure rather than artefacts of model specification.

Accordingly, several intercept (constant) terms were removed during stepwise collinearity reduction, including those associated with volume, sperm concentration, total motility, total estimated volume, total real added volume, and, in earlier rounds, endosmosis. These terms exhibited extreme VIF inflation due to strong linear dependence on both their corresponding polynomial derivatives (b₂ and b₃ terms) and on closely related seminal quality traits, a pattern consistent with known behavior of polynomial expansions and biologically coupled predictors^37^. This behavior is biologically expected given the structure of the predictors and the physiology of semen production.

Indeed, ejaculate volume and sperm concentration act as scale-setting traits, such that individuals with higher baseline levels tend to show proportionally larger dynamic variation across ejaculates, producing near-linear dependence between constant and polynomial components^38^. Similarly, total motility and dose-related traits are composite measures functionally downstream of volume, concentration, and progressive motility, causing their baseline values to be largely explained by these underlying variables^27^. Endosmosis, reflecting membrane integrity, is tightly linked to motility and sperm morphology, and its baseline level constrains higher-order temporal variation. Consequently, inclusion of both intercept and dynamic terms for these traits repeatedly encoded the same biological gradients, producing severe multicollinearity. Importantly, removal of intercept terms does not imply loss of biological information; rather, the variance they represent is retained through correlated baseline or dynamic predictors that remained in the final model, consistent with best practices for reducing redundant predictors in multivariate biological analyses^39^. The resulting reduced predictor set therefore preserves biologically meaningful variation while eliminating redundant representations, enabling stable estimation and interpretable canonical structures.

Semen quality is influenced not only by genetic background but also by intrinsic factors such as age, body development, and semen collection frequency^40,41^. In the present study, the time variable in all regression models was defined as the animal’s age at each semen collection, with each observation corresponding to the age (in months) at the moment of sperm sampling.

Accordingly, the regression coefficients describe age-related biological dynamics rather than generic temporal trends. The constant coefficient represents baseline trait values at the reference age (e.g., initial sperm concentration), the quadratic coefficient (b₂) captures curvature in age-dependent trajectories (maturation-related changes in ejaculate volume), and the cubic coefficient (b₃) models longer-term, nonlinear age effects, reflecting complex physiological processes such as endosmosis and progressive sperm membrane functionality.

Although correlations among coefficients were generally weak, important associations emerged. For example, the strong positive correlation between the Sperm Concentration constant and b3 Endosmosis indicates that higher baseline sperm concentration is linked to sustained membrane integrity over time. This observation is consistent with the notion that denser ejaculates provide a protective environment that maintains osmotic balance, supporting motility and overall sperm viability^42,43^. Conversely, a negative relationship between the Progressive Motility constant and b2 Total Estimated Volume suggests that higher baseline progressive motility is associated with declining ejaculate volume over time, reflecting dilution effects commonly reported in the literature^44^.

A particular focus on cubic coefficients (b3) provides insight into long-term nonlinear dynamics of semen traits, which are often overlooked in simpler linear or quadratic models. Biologically, b3 captures the curvature associated with processes that do not follow simple monotonic trends but instead exhibit delayed or cumulative effects over multiple ejaculates or across the reproductive lifespan of the sire.

The positive correlation of b3 Endosmosis with baseline sperm concentration suggests that genotypes producing dense ejaculates also sustain long-term membrane integrity. This may reflect the ability of seminal plasma proteins, including caseins or casein-derived peptides, to protect sperm against oxidative stress and osmotic shock over repeated ejaculates^45^^,46^.

Cubic terms can reveal compensatory patterns in semen traits, such as initial declines in volume or motility followed by stabilization or minor recovery in later collections. This is particularly relevant for evaluating sires over time, as early measurements alone may underestimate lifetime reproductive potential.

Nonlinear cubic dynamics can reflect biological trade-offs, such as the balance between ejaculate volume and sperm motility. For example, a negative b3 in progressive motility paired with a positive b3 in ejaculate volume could indicate that while motility declines slowly over time, the overall ejaculate yield is maintained, potentially through seminal plasma buffering or adaptive energy allocation.

Examining αs1-casein genotypes, cubic coefficients helped differentiate genetic profiles of semen quality. The BE genotype was associated with low Sperm Concentration constants and negative b2 Volume, highlighting declining ejaculate volumes with age, and typically exhibited negative or low b3 coefficients, suggesting poor long-term stability in motility or membrane function. In contrast, EE genotypes combined high Progressive Motility constants with positive b3 Endosmosis coefficients, indicating that superior baseline motility is maintained across repeated ejaculates. AB genotypes showed high Volume constants but low Sperm Concentration constants and negative b3 for motility traits, illustrating a volume–quality trade-off. AE and BB genotypes presented intermediate cubic profiles, suggesting some ability to sustain ejaculate volume and concentration over time, albeit with more variability.

The κ-casein CHAID decision tree reinforced these patterns. AA bucks consistently showed favorable Sperm Concentration constants and positive b3 values for both motility and acrosome integrity, reflecting sustained sperm functionality. BB bucks, although associated with high Volume constants, exhibited low Sperm Concentration constants and negative b3 for motility traits, highlighting a long-term decline in sperm quality despite high ejaculate yield. Such findings illustrate that cubic coefficients are particularly informative for identifying sires whose semen traits are robust over time, rather than merely performing well at a single collection. When αS1- and κ-casein genotypes were considered jointly, distinct genotype-combination–specific profiles of semen quality emerged, highlighting heterogeneity in how these loci relate to seminal characteristics, as previously suggested for dairy and reproductive traits in other ruminants^47^. Combinations such as EE–AA and AE–AA were associated with high baseline progressive motility and sperm concentration, together with positive higher-order terms (*b*_3_ Endosmosis), suggesting favorable baseline levels alongside sustained dynamic performance over time. Similar genotype-dependent patterns linking casein variation to sperm quality traits have been reported elsewhere, although such interpretations remain tentative when based on rare genotype combinations^48^. Intermediate combinations, including AB–AB and BB–AB, displayed more balanced profiles characterized by stable ejaculate volumes and intermediate semen quality parameters, a pattern consistent with earlier observations of trade-offs between robustness and performance in breeding populations^49^. In contrast, the BB–BB combination was associated with increased semen volume but comparatively lower or declining motility and concentration, supporting the notion of a trade-off between semen yield and qualitative attributes, as reported in other livestock species^50^. Notably, the more frequently observed BE–AB combination showed consistent intermediate-to-high semen quality across multiple traits, indicating a relatively stable phenotypic pattern within the studied population^47^. Overall, these findings underscore the presence of genotype-combination–specific semen quality profiles and identify combinations that merit further investigation, particularly through fertility outcomes, longitudinal analyses, and validation in independent populations.

While these genotype-combination–specific profiles provide biologically coherent insights into how αS1- and κ-casein variation may relate to semen quality, their practical value ultimately depends on the extent to which such patterns can be captured and generalized by predictive classification models. In this context, evaluating not only the direction of discriminant associations but also their stability, misclassification risk, and sensitivity to genotype frequency is essential. Differences in predictive robustness between loci may therefore reflect both underlying biological relevance and the statistical constraints imposed by class imbalance and overlapping phenotypic distributions, particularly for low-frequency genotype combinations. These considerations motivate a locus-specific assessment of model performance, placing the observed discriminant patterns within a broader framework of predictive reliability.

In this regards, CHAID analysis revealed clear contrasts in predictive performance between κ-casein and αS1-casein models. For κ-casein, the relatively high misclassification risk (0.429; SE = 0.079), corresponding to an overall accuracy of approximately 57.1%, indicates limited discriminatory power, in line with previous reports of modest genotype resolution for this locus^51^. By contrast, the αS1-casein model exhibited a substantially lower risk (0.325; SE = 0.074) and higher classification accuracy (≈ 67.5%), reflecting a stronger ability to distinguish genotypic classes, consistent with earlier findings highlighting the greater functional relevance of αS1-casein variation^48^. For both loci, identical risk estimates under resubstitution and cross-validation indicate stable tree structures and no evidence of overfitting, supporting the robustness of the classification framework^49^. Collectively, these results suggest that the explanatory variables included in the analysis are more informative for αS1-casein variation than for κ-casein, while also reinforcing that discriminant associations—particularly for low-frequency genotype combinations—should be interpreted as exploratory rather than definitive^50^.

The biological interpretation of the discriminant models is directly supported by the structure and weighting of the standardized coefficients defining the canonical functions (F1 and F2) for both αS1- and κ-casein genotypes. In both loci, the first discriminant function (F1) is characterized by strong positive loadings of constant sperm concentration (αS1: +0.610; κ: +0.591), constant acrosome integrity (αS1: +0.269; κ: +0.373), and constant ejaculate volume components (real total added volume), together with consistently negative second-order coefficients for acrosome integrity (b2) (αS1: −0.623; κ: −0.706). This coefficient pattern indicates that F1 primarily reflects a baseline semen quality axis, integrating constitutive spermatogenic output and structural sperm membrane integrity, while penalizing pronounced short-term curvature in acrosomal stability, consistent with established relationships between membrane integrity, acrosomal function, and fertilizing capacity^27,52^. Accordingly, genotype separation along F1 is driven mainly by stable, time-independent semen characteristics rather than by transient fluctuations.

In contrast, the second discriminant function (F2) in both casein loci is dominated by dynamic regression components, most notably the strong negative coefficients for b2 volume (αS1: −0.910; κ: −0.915) and the large positive coefficients for non-linear endosmosis responses (b3) (αS1: +0.725; κ: +0.807). The simultaneous presence of positive constant acrosome integrity and negative b2 acrosome integrity in F2 further indicates that this axis captures genotype-specific differences in the temporal adaptability of sperm membrane function across collections, rather than absolute membrane quality, aligning with known plasticity of sperm functional traits under physiological modulation^53^. The inverse signs of volume-related and concentration-related coefficients in F2 also suggest contrasting ejaculate allocation strategies, whereby changes in ejaculate volume dynamics are coupled with shifts in sperm concentration and membrane resilience, a pattern consistent with theoretical models of ejaculate partitioning and sperm investment^54^.

Taken together, the opposing coefficient structures of F1 and F2 demonstrate that casein genotypes are associated with distinct combinations of static and dynamic semen quality components. The discriminant functions therefore support a biological model in which αS1- and κ-casein genotypes modulate not only constitutive semen quality but also the non-linear regulation and stability of sperm function over time, reinforcing the interpretation of casein effects as shaping complex, multivariate reproductive phenotypes rather than isolated seminal traits^39^.

Biologically, these genotype–trait associations may reflect shared molecular pathways linking casein expression to reproductive function. Genes such as IGF1, essential for spermatogenesis and testicular development, also influence milk composition and casein content^55–57^. Similarly, the JAK2-STAT5 pathway, which regulates casein expression, also contributes to antioxidant defenses and testicular function^58,59^. Caseins themselves confer antioxidant protection, stabilizing both micelles and seminal plasma, which may explain why AA genotypes in κ-casein are linked to better acrosome integrity and motility^45,46,60^. The cubic coefficients, in particular, appear to reflect these long-term protective effects, capturing how certain genotypes maintain semen quality trajectories over the reproductive lifespan of bucks.

## Methods

### Sample and study conditions

**Fig. 8:**
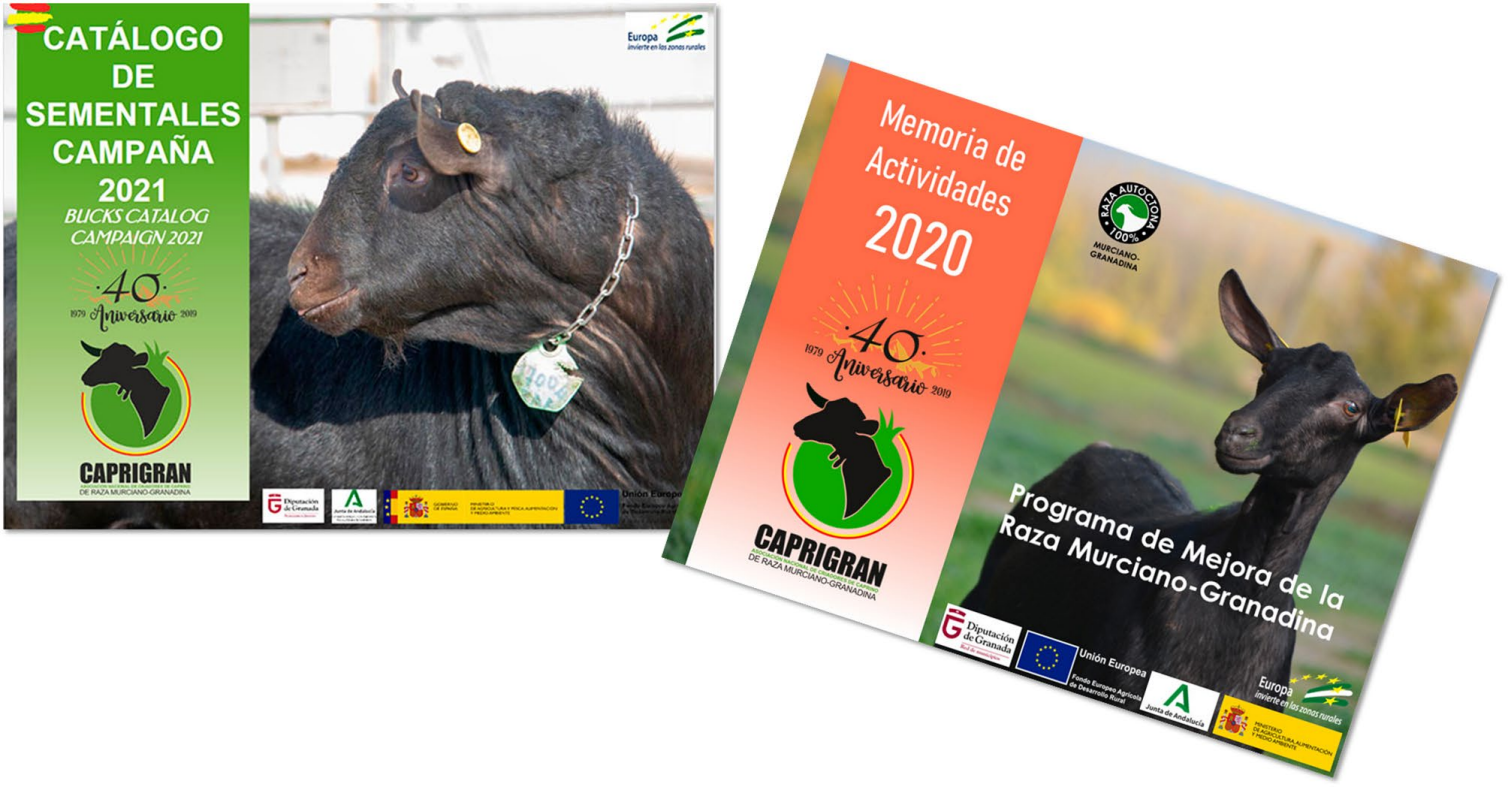
Murciano-Granadina Sire Catalogues from 2020 and 2021.

The study was conducted over a ten-year period, from January 2010 to December 2019, and involved 115 Murciano-Granadina bucks born between November 2003 and December 2018 (Fig. 8). During this interval, a total of 6,868 ejaculates were collected and evaluated. All animals were part of the official breeding program for the breed and were selected based on proven fertility records.

**Fig. 9:**
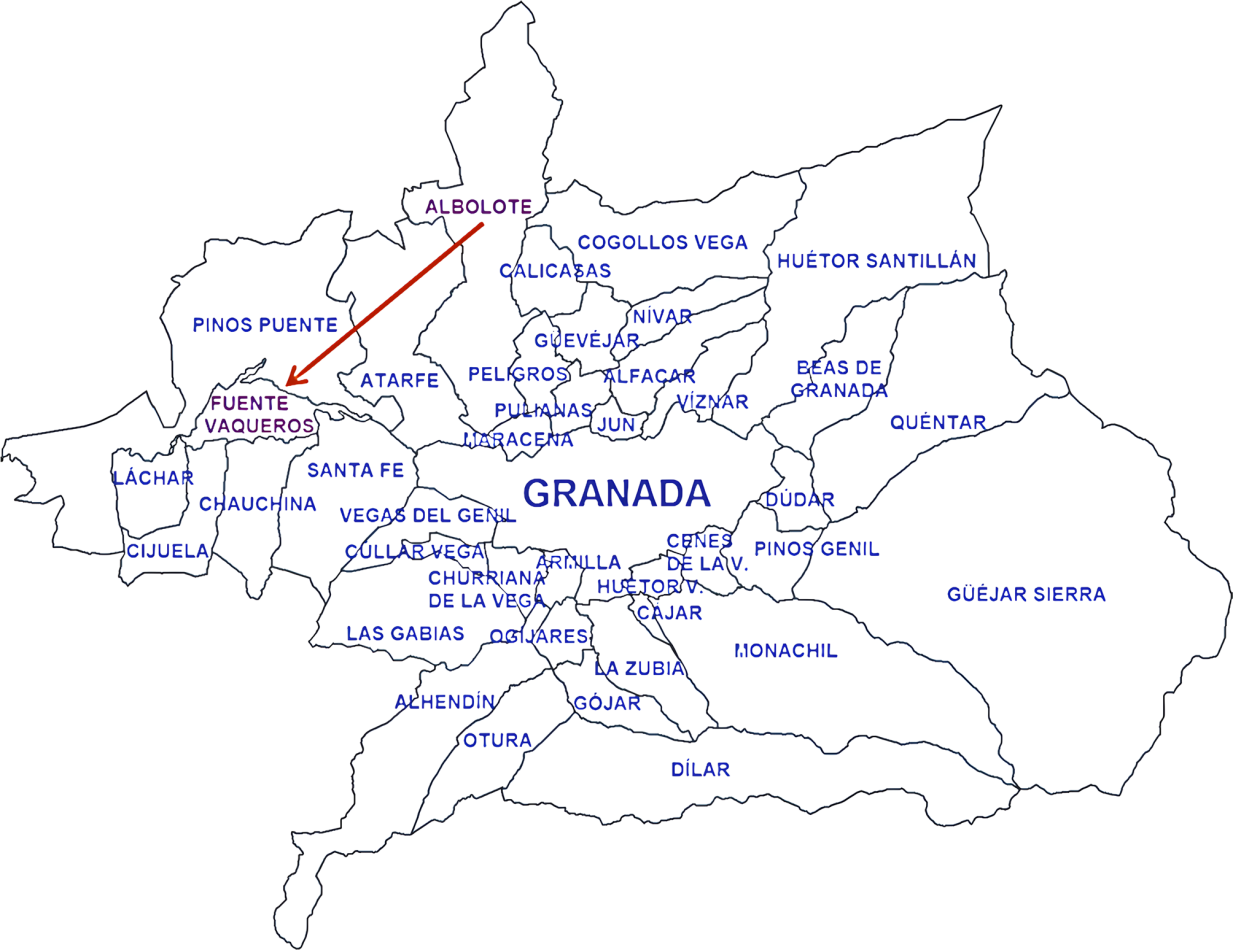
Relocation of CAPRIGEN (Goat Biotechnology Center) from Albolote to Fuente Vaqueros, shown on a map of the municipalities within the Granada Plain (Vega de Granada).

Initially, bucks were housed at CAPRIGEN, the Andalusian Center for Goat Selection and Genetic Improvement in Albolote (Granada, Spain). In 2014, the animals were transferred to newly established facilities in Fuente Vaqueros, within the Santa Fe municipality of Granada (Fig. 9). Both centers are situated in the fertile plains of the Vega de Granada, a region characterized by a continental Mediterranean climate with wide daily temperature fluctuations often exceeding 20 °C. The area experiences an average annual rainfall of 420 mm, mainly concentrated in autumn and winter, with 6–9 frost days per season during the colder months.

Throughout the study, uniform husbandry conditions were maintained. The feeding regime consisted of 0.5 kg of commercial concentrate per animal per day, supplemented with ad libitum hay. Supplementary Table S6 summarizes the detailed ingredient composition, additives, and analytical nutrient profile of the standardized diet provided to all animals throughout the study period. Fresh drinking water and mineral blocks were continuously provided to ensure adequate nutrition and minimize dietary variability, thereby reducing potential confounding effects on reproductive performance.

### Semen collection and analysis

Semen was collected using an artificial vagina and immediately placed in a water bath at 37 °C to maintain physiological conditions. Ejaculate volume (mL) was measured in calibrated tubes, mass motility was assessed on a 0–5 scale using a phase-contrast microscope at 40× magnification (Olympus, Tokyo, Japan), and sperm concentration was determined with a photometer (Accurread, IMV Technologies, France). For computer-assisted sperm analysis, samples were diluted in INRA 96 extender to a final concentration of 20 × 10^6^ spermatozoa/ml and incubated at 37 °C for 10 min. Aliquots of 5 µl were then placed on pre-warmed slides with 22 × 22 mm coverslips and analyzed using ISAS v1.2 software (Proiser, Valencia, Spain), recording four random fields per sample to estimate total motility (TM, %) and progressive motility (PM, %). Acrosome integrity (% of intact acrosomes) was evaluated by preparing smears with 10 µl of semen, fixing them in methanol for 1–2 min, and examining 200 spermatozoa per slide under 100× oil immersion, with the proportion of intact acrosomes calculated accordingly. Plasma membrane functionality was assessed using the hypo-osmotic swelling test (HOST) following Jeyendran et al. (1984), in which 10 µl of semen was mixed with 100 µl of hypo-osmotic solution (100 mOsm/kg; 1.351 g fructose + 0.735 g sodium citrate in 100 ml bidistilled water), incubated at room temperature for 30 min, and fixed in 2% glutaraldehyde. At least 200 spermatozoa per sample were observed at 400× magnification, with coiled tails indicating functional plasma membranes. All analyses were conducted under standardized laboratory conditions by trained personnel to ensure consistency and minimize observer-related variation.

### αS1- and κ-Casein genotyping

Molecular characterization of the casein complex in the Murciano-Granadina breeding programme is routinely performed using a panel of 48 single nucleotide polymorphisms (SNPs) distributed across the main casein genes, of which 25 are specific to the αS1-casein and κ-casein loci. This SNP panel enables the unambiguous determination of αS1-casein and κ-casein genotypes, which are systematically recorded and published in the official Murciano-Granadina Sire Catalogue. Consequently, all bucks included in the present study had been genotyped for αS1-casein and κ-casein as part of the routine breeding programme prior to this study. All animals were part of the official breeding programme for the breed and were selected based on proven fertility records. As a result, the dataset represents the population of active artificial insemination sires rather than an experimentally selected subset; while this may limit extrapolation to unselected or non-breeding males, it ensures that genotype–phenotype relationships are evaluated under real breeding conditions, which is the intended scope of the study. 

Genomic DNA is routinely extracted using a modified salting-out protocol^61^. Method validation was conducted on 16 unrelated goats randomly selected from the herdbook. SNPs located in promoter regions, 3′ untranslated regions (UTR), and polymorphic exons were defined as described by^62^. Target regions were amplified using a Platinum High-Fidelity PCR kit (Life Technologies, CA, USA), and sequencing was performed by Macrogen Inc. (Korea). Chromatograms were inspected and aligned using MEGA7, with variant annotation supported by the Ensembl Genome Browser (release 97;^63^).

Genotyping of the 48 SNPs was carried out using a Kompetitive Allele Specific PCR (KASP) assay (LGC Limited, UK), and allelic discrimination was performed with KlusterCaller software (LGC Limited, UK). The observed heterozygosity (~ 40%) confirmed that the selected SNP panel provided adequate control of population stratification^64^. Minor allele frequencies (MAF) were estimated using PLINK v1.90, allowing discrimination between common and rare variants (MAF < 0.05)^65^.

Linkage disequilibrium (LD) across the casein complex was assessed using HaploView, calculating both D′ and r^2^ statistics for all SNP pairs. Total locus length and interlocus distances were determined following the approach of^66^. Detailed LD metrics are reported in Supplementary Table S7.

For the purposes of this study, genotypic information for αS1-casein and κ-casein was retrieved directly from routine breeding programme records; no additional genotyping or targeted sampling strategy was required. Only animals with complete and validated genotypic and phenotypic records were included in the analyses.

**Fig. 10:**
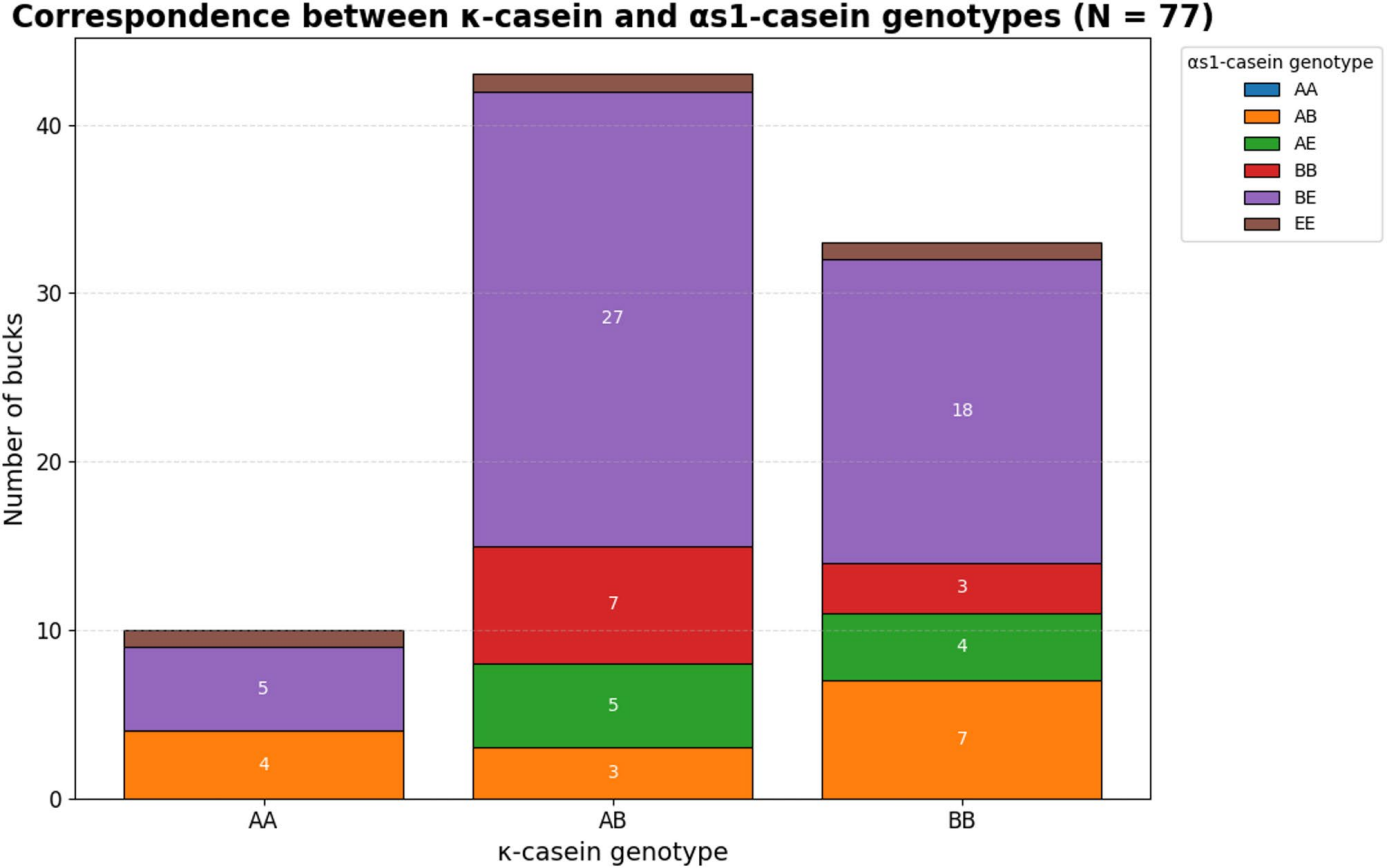
Stacked bar chart showing the combinations of κ-casein and αs1-casein genotypes found in each animal (*n* = 115). Bars represent κ-casein genotypes, and stacked segments indicate the number of animals carrying each αs1-casein genotype within κ-casein classes.

The combinations of κ-casein and αS1-casein genotypes observed in each animal were summarized descriptively to characterize their joint distribution within the population. Genotype combinations were tabulated and visualized to show the frequency of each κ-casein × αS1-casein profile, without inference or modeling. This visualization allows identification of dominant and rare multilocus profiles and facilitates interpretation of subsequent classification and association analyses. The resulting distribution is presented in Fig. 10.

### Statistical analysis

#### Parametric assumption testing

Analytical parametric assumptions were systematically evaluated prior to model fitting, CDA, and CHAID analyses at two analytical levels. On the one hand, ejaculates (*n* = 6,868) were treated as the statistical unit for model fitting and longitudinal analyses, whereas on the other hand, bucks (*n* = 115) were treated as the statistical unit for canonical discriminant analysis (CDA) and CHAID models, with each animal contributing a single observation derived from individual-level cubic regression coefficients sets (b0, b1, b2, b3).

Normality was assessed using the Shapiro–Francia W′ test, which is suitable for moderate to large sample sizes (50–2500) and exhibits increased sensitivity to upper-tail departures from normality. Accordingly, Shapiro–Francia was applied at both the ejaculate level (*n* = 6,868) and the buck level (*n* = 115; optimal *n* ≥ 100), given departures from normality were biologically plausible given that animals were housed at the Andalusian Center for Goat Selection and Genetic Improvement and had been pre-selected for superior standardized semen quality.

Given the large number of ejaculates, results from formal normality testing at the ejaculate level were interpreted cautiously, as such tests are known to be overly sensitive in large samples. Distributional assumptions were therefore primarily evaluated using graphical diagnostics (Q–Q plots), with the Shapiro–Francia test applied descriptively rather than as a strict decision criterion. All normality assessments were performed using the *Test and Distribution Graphics* routine in Stata version 15.0 (StataCorp, College Station, TX, USA).

Homogeneity of variances was evaluated using Levene’s test, conducted through the *Explore* procedure in the *Descriptive Statistics* package of SPSS Statistics version 25.0 (IBM Corp., Armonk, NY, USA).

The unit of analysis varied according to the modeling framework. For descriptive and longitudinal analyses, ejaculates were treated as the statistical unit (*n* = 6868 ejaculates). In contrast, for canonical discriminant analysis (CDA) and CHAID models, the statistical unit was the buck (*n* = 115), as these analyses were based on individual-level cubic regression coefficients, yielding one observation per animal.

First-order autocorrelation of residuals was evaluated using the Durbin–Watson test (Durbin, 1970), conducted through the *Linear…* subroutine of the *Regression* procedure of the *Analyze* package in SPSS Statistics version 25.0 (IBM Corp., Armonk, NY, USA). The DW statistic ranges from 0 to 4, with values close to 2 indicating the absence of autocorrelation, values below 2 suggesting positive autocorrelation, and intermediate values considered inconclusive. This diagnostic is appropriate for ordered data, particularly time-dependent observations such as longitudinal measurements of semen or testicular traits.

Because ejaculates were explicitly treated as a time series indexed by age at collection, temporal ordering was biologically meaningful within each buck. Accordingly, the Durbin–Watson test was applied at the individual-buck level, evaluating whether first-order autocorrelation was present in the age-ordered residuals of each animal’s series. This application is appropriate because DW assesses serial dependence within a single ordered sequence, and in this study each buck contributed its own short longitudinal series of ejaculates^37^. Although the data have a hierarchical structure, the DW test was not intended to model between-buck clustering; rather, it served as a diagnostic for within-buck temporal autocorrelation, which is the only dependency structure the statistic is designed to detect^67^. Hierarchical and between-animal variability were addressed separately through the study design and multivariate modeling framework, while the DW test confirmed that temporal autocorrelation did not confound the age-based regression components^68^.

Homoscedasticity, a key assumption of ordinary least squares regression, was further examined through visual inspection of scatterplots of residuals versus standardized predicted values, where non-uniform dispersion was interpreted as evidence of heteroscedasticity. Residuals were calculated as the difference between observed and predicted values.

Outlier screening was performed using the ROUT method in the *Identify Outliers* routine of GraphPad Prism version 9.0 (GraphPad Software, San Diego, CA, USA). This approach combines robust regression with outlier detection while controlling the false discovery rate (FDR) via a Q coefficient, which was set at 1% in the present study to apply a highly conservative threshold and minimize false-positive identification^69^.

Seminal Quality Parameters Regression Approach Determination.

For modeling seminal quality dynamics, a total of eleven models—one linear and ten non-linear—were applied to describe the evolution of seminal traits across the 115 bucks included in the study (Supplementary Table S8). Semen quality parameters were regressed against the age of each buck at semen collection individually, with age used as the time index and each observation corresponding to a semen collection event for a given buck.

The Curve Estimation task within the Regression procedure of SPSS was used to iteratively define parameter bounds (b_0_, b_1_, b_2_, b_3_, b_4_) via the Levenberg–Marquardt iteration method, continuing until convergence at a tolerance criterion of 10⁻⁸ for the error sum of squares between successive iterations, thereby ensuring stable and precise parameter estimates. These estimates were then used to implement automated model-fitting protocols for each buck and seminal trait individually.

#### Model choice

Model selection was based on multiple criteria, including Residual Sum of Squares (RSS), Mean Squared Prediction Error (MSPE), Adjusted R^2^ (Adj. R^2^) and its standard deviation across individuals, Akaike Information Criterion (AIC), corrected AIC (AICc), and Bayesian Information Criterion (BIC). RSS quantified unexplained variance, while cross-validated MSPE evaluated error variation with reduced sensitivity to the number of parameters in limited samples. Adj. R^2^ accounted for model complexity, penalizing overfitting while measuring predictive accuracy. AIC and AICc were calculated to compare explanatory power across models, with AICc providing a more accurate assessment in datasets where the ratio of observations to parameters was limited (N/K < 40). BIC was used to evaluate predictive potential, penalizing models with additional parameters similarly to Adj. R^2^, thereby aiding in the selection of models that balanced explanatory and predictive performance.

Once the regression model describing seminal quality dynamics as a function of age had been selected, models were fitted individually for each buck. The coefficients derived from these individual regression fits were then used as explanatory variables in the canonical discriminant analysis (CDA) and CHAID decision trees, as described in subsequent sections of the study.

#### Canonical discriminant analysis (CDA)

Canonical discriminant analysis (CDA) was applied to classify semen samples using the αS1- and κ-casein genotypes as the grouping factor to evaluate whether linear combinations of semen quality traits could discriminate among genotypic groups. αS1-casein genotypes were categorized as AA, AB, AE, BB, BE, and EE, while κ-casein genotypes were AA, AB, and BB, in two separate analyses respectively.

Forward stepwise multinomial logistic regression was used for variable selection. Class priors were specified to be proportional to observed group sizes using SPSS Version 26 (IBM Corp., Armonk, NY, USA) to account for unequal sample representation. In SPSS multinomial logistic regression, prior probabilities for outcome categories may be specified by the user or estimated from the data; in the present study, priors were estimated from observed group frequencies by selecting *Estimate from data*. This assigns each outcome category a prior probability proportional to its empirical prevalence and incorporates class prevalence directly into the likelihood function used for parameter estimation and likelihood-ratio–based variable selection, without modifying the underlying data.

Forward and backward stepwise procedures identified the same set of predictors; therefore, progressive forward selection was retained for computational efficiency. Model selection was based on the likelihood-ratio criterion implemented in SPSS, with variables entering and remaining in the model according to changes in − 2 log-likelihood.

Model fitting employed maximum likelihood estimation, likelihood-ratio–based variable entry and removal criteria (entry at α = 0.05; removal at α = 0.10), standard indicator (dummy) coding of categorical predictors, a maximum of 20 iterations per step, and a convergence threshold based on changes in − 2 log-likelihood. Missing data were handled by listwise deletion, and no interaction terms were specified. These settings ensured transparency and full reproducibility of the variable selection procedure.

Variable selection was conducted for screening purposes rather than final inference or prediction, in a context involving a relatively large set of potentially correlated candidate explanatory variables. Importantly, these variables were not raw semen quality measurements but coefficients derived from individual buck-level longitudinal regression models, which substantially reduced data dimensionality and alleviated collinearity prior to stepwise selection. Within this framework, stepwise multinomial logistic regression provided a pragmatic and transparent approach to identify a parsimonious subset of informative coefficients for subsequent multivariate analyses.

The variables retained through this screening step were not interpreted in isolation. Their relevance was subsequently evaluated using independent multivariate approaches—canonical discriminant analysis (CDA) and CHAID decision trees—which rely on different assumptions and classification principles. Consistent patterns across these complementary methods provided implicit validation of the selected variables and reduced the likelihood that selection was driven by overfitting or method-specific artefacts.

#### Explanatory variables

The semen quality traits analyzed in this study included total estimated volume (mL), total doses (n), real added total volume (mL), real doses (n), volume per dose (mL), sperm concentration (×10^6^ spermatozoa/mL), total motility (%), progressive motility (%), endosmosis (%), and Acrosome Integrity/Intact acrosomes (%). These traits were modeled using cubic regression, with the regression coefficients (b0, b1, b2, and b3) serving as the explanatory variables Each coefficient describes a distinct aspect of the semen parameter dynamics: b0 represents the baseline level, reflecting the inherent or average trait value independent of trends; b1 quantifies the linear effect, capturing monotonic increases or decreases over the measured range; b2 describes the curvature (quadratic) effect, highlighting acceleration or deceleration in the trait’s progression; and b3 characterizes the cubic (complex) trend, representing subtle inflections or non-linear fluctuations in trait dynamics that cannot be captured by linear or quadratic terms alone. Collectively, these coefficients provide a detailed, biologically meaningful decomposition of seminal quality traits, enabling the assessment of how casein genotypes modulate both simple and complex patterns in semen production and sperm functionality.

#### Sample size considerations

The ratio of observations to predictors was sufficient to ensure robust Canonical Discriminant Analysis (CDA). Guidelines recommend a minimum of 20 observations per 4–5 predictors^70^, and the dataset used in this study exceeded this requirement by a factor of four to five, providing adequate statistical power.

#### Multicollinearity assessment

Stepwise reduction was applied as a pragmatic strategy to mitigate severe multicollinearity while preserving the interpretability of biologically meaningful predictors^71,72^. Unlike orthogonalization or penalized approaches (e.g., PCA, ridge, or lasso), this strategy allows direct interpretation of canonical loadings in the original variable space, which is essential for biological inference^39^. Multicollinearity among predictors was evaluated using variance inflation factors (VIF) and tolerance values, with VIF calculated as

$$\mathrm{VIF}=\frac{1}{1-{R}^{2}},$$.

where ${R}^{2}$is the coefficient of determination obtained by regressing each predictor against all remaining predictors. A conservative threshold of VIF ≤ 5 was adopted to ensure acceptable predictor independence and numerical stability.

Extremely inflated VIF values (> 70,000) observed in the initial model reflected structural redundancy among polynomial coefficients and strongly correlated semen quality traits. Stepwise reduction was therefore used to progressively eliminate redundant predictors until collinearity was reduced to acceptable levels. This procedure was intended to stabilize model estimation rather than to perform inferential variable selection, and the final retained predictor set is explicitly reported (Supplementary Table S2). Sensitivity analyses across successive reduction rounds showed consistent canonical structures, indicating robustness of the retained solution.

Alternative approaches such as mixed-effects modeling were not adopted because the primary source of dependency in the data arose from functional and mathematical relationships among predictors, rather than from hierarchical or clustered sampling structures that mixed models are designed to address^67^. Moreover, introducing random effects would not resolve linear dependence among polynomial terms and would substantially complicate interpretation of canonical loadings^37^. Given the study’s exploratory objective and emphasis on biological interpretability, a reduced fixed-effects predictor set was therefore preferred^73^.

#### Canonical correlation dimensions

The maximum number of canonical correlations was equal to the number of variables in the smaller set. Canonical correlations greater than or equal to 0.30 were considered meaningful, as they explain approximately 10% of the shared variance between sets.

#### CDA model adequacy and reliability

To evaluate the adequacy and robustness of the canonical discriminant analysis (CDA), several complementary statistical criteria were applied. Bartlett’s test was used to assess the overall significance of the canonical functions, while Wilks’ lambda quantified the proportion of unexplained variance and the discriminatory power of the model. Pillai’s Trace Criterion was included as a more robust multivariate test, particularly suitable under potential deviations from multivariate normality. In addition, false discovery rate (FDR) correction was applied to control for multiple testing and reduce the risk of type I error. Together, these measures provide a comprehensive assessment of model significance, stability, and reliability.

#### Bartlett’s test

Bartlett’s test in canonical discriminant analysis evaluates whether each successive canonical function explains a statistically significant amount of discrimination between groups by testing the null hypothesis that the remaining canonical correlations (and their associated eigenvalues) are equal to zero. Accordingly, a significant Bartlett’s statistic (*p* < 0.05) indicates that the corresponding canonical function contributes to group separation beyond random expectation.

Bartlett’s test of sphericity was performed in XLSTAT (Addinsoft Pearson Edition 2014, Addinsoft, Paris, France) and was applied to verify that the correlation matrix significantly differed from an identity matrix (*p* < 0.05), confirming suitability for CDA.

#### Wilks’ lambda test

Wilks’ lambda was performed in XLSTAT (Addinsoft Pearson Edition 2014, Addinsoft, Paris, France) and was used to evaluate univariate contribution, with values closer to 0 indicating stronger discrimination between groups. Significance was assessed using chi-square tests (*p* < 0.05).

#### Pillai’s trace criterion and false discovery rate (FDR)

Pillai’s Trace criterion was computed in XLSTAT (Addinsoft Pearson Edition 2014, Addinsoft, Paris, France) to assess multivariate effects while providing robustness against potential deviations from multivariate normality and homoscedasticity. In addition, false discovery rate (FDR) control was applied to univariate p-values using the Benjamini–Hochberg procedure, implemented post hoc in Microsoft^®^ Excel^®^ for Microsoft 365 (MSO, Version 2511, Build 16.0.19426.20218, 64-bit, English). For each family of related tests, p-values were first verified to be numeric, then sorted in ascending order and assigned a rank (*i*), with the smallest p-value assigned *i* = 1 and the total number of tests denoted as *m*. For each ranked p-value, the Benjamini–Hochberg critical value was calculated as $(i/m)\cdot q$, where *q* represents the chosen FDR level (0.05), and tests satisfying $p\le(i/m)\cdot q$were considered significant after FDR correction. In addition, FDR-adjusted p-values (*q*-values) were computed using the expression $p\cdot m/i$, with monotonicity enforced by replacing each adjusted value with the minimum of its own value and those of all higher-ranked tests, thereby ensuring a non-decreasing sequence of *q*-values. All FDR calculations were performed independently for each genotype-specific test family. Supplementary Material S1 provides a detailed description of the implementation of the false discovery rate (FDR) correction using the Benjamini–Hochberg procedure in Microsoft Excel, ensuring transparency and reproducibility of the multiple-testing control applied in this study.

Canonical discriminant analysis (CDA) was performed in XLSTAT (Addinsoft Pearson Edition 2014, Addinsoft, Paris, France), where the method is based on the decomposition of the between-group (B) and within-group (W) covariance matrices and the solution of the generalized eigenvalue problem $\mathrm{B}\mathrm{v}=\lambda\mathrm{W}\mathrm{v}$. Canonical discriminant functions, along with canonical coefficients, standardized coefficients, and structure loadings, were derived directly from this decomposition. Canonical standardized coefficients and structure loadings were used to assess the contribution of each variable to the discriminant functions. All variables were included in the estimation of the canonical discriminant functions; however, interpretation focused on variables with substantive structure loadings and variables with absolute structure loadings ≥ |0.40|, consistent with common practice. Pairwise group separation was quantified using squared Mahalanobis distances, calculated as:

$${\rm{Di}}{{\rm{j}}^{\rm{2}}} = {\rm{ }}\left( {{\rm{Yi }} - {\rm{ Yj}}} \right){\rm{ }} \times {\rm{ CO}}{{\rm{V}}^{ - {\rm{1}}}} \times {\rm{ }}\left( {{\rm{Yi }} - {\rm{ Yj}}} \right)$$

where Dij^2^ is the squared distance between populations i and j, COV^-1^ is the inverse of the pooled covariance matrix, and Yi and Yj are the group means. Distances were converted into Euclidean metrics and visualized using UPGMA clustering dendrograms in Dendrogram App^74^.

#### Effect size-based discriminant contributions of Casein genotypes

Effect sizes for the discriminant contribution of individual semen-trait regression components were quantified using partial eta squared (ηp^2^) derived from the univariate F-tests of equality of group means (based on Wilks’ Lambda) associated with each predictor. Partial eta squared was calculated as ηp^2^ = (F × df_1_)/(F × df_1_ + df_2_), where F denotes the observed F statistic and df_1_ and df_2_ represent the numerator and denominator degrees of freedom, respectively.

This metric estimates the proportion of between-genotype variance explained by each regression component independently of sample size. To characterize the uncertainty of effect-size estimates, 95% confidence intervals (CI) for ηp^2^ were obtained using the noncentral F distribution, with lower and upper bounds computed from the corresponding noncentrality parameters. Effect sizes were interpreted using conventional thresholds (small: ηp^2^ < 0.06; moderate: 0.06–0.14; moderate–large: 0.14–0.26; large: ≥ 0.26).

#### CDA function leave-one-out cross-validation

Classification accuracy was quantified as hit ratios, representing the percentage of samples correctly classified into their genotypic group. Leave-one-out cross-validation (LOOCV) was used to test model reliability. Press’s Q statistic was calculated using Press’ Q Statistic Calculator v. 1.0^74^ as:

$${\rm{Q }} = {\rm{ }}\left( {{{\left( {{\rm{n }} - {\rm{ }}\left( {{\rm{n' }} \times {\rm{ K}}} \right)} \right)}^{\rm{2}}}} \right)/\left( {{\rm{n }} \times {\rm{ }}\left( {{\rm{K }} - {\rm{ 1}}} \right)} \right)$$

where n is the total number of observations, n′ is the number of correctly classified observations, and K is the number of groups. Q values exceeding the chi-square critical value of 6.63 (df = 1, *p* < 0.01) indicated classification accuracy at least 25% above chance.

#### CHAID decision tree data mining

To complement CDA, a Chi-squared Automatic Interaction Detection (CHAID) decision tree was applied to classify and predict genotypic groups based on discretely categorized semen traits. Node splitting was performed using Chi-square tests with Bonferroni-adjusted significance levels (*p* < 0.05). To avoid overfitting, tree pruning was implemented.

#### CHAID decision tree construction and pre-pruning criteria

Classification trees were constructed using the Chi-square Automatic Interaction Detection (CHAID) algorithm as implemented in XLSTAT (Addinsoft Pearson Edition 2014, Addinsoft, Paris, France). Tree growth and model complexity were controlled exclusively through pre-pruning (early stopping) criteria, with no post-pruning procedures applied. The maximum tree depth was fixed at five levels, and node size constraints required a minimum of two observations in parent nodes and one observation in child nodes for a split to be considered.

Node splitting and category merging were based on Pearson’s chi-square statistic, with the significance level set at 5% for both splitting and merging operations. A Bonferroni correction was applied to adjust significance values for multiple testing. Category redivision within nodes was authorized, allowing previously merged categories to be re-split when statistically justified. The CHAID merge and split thresholds were both set to 5%, in accordance with the default XLSTAT configuration (Addinsoft Pearson Edition 2014, Addinsoft, Paris, France).

For quantitative explanatory variables, discretization was performed using univariate clustering into 10 intervals prior to tree construction. Model estimation was allowed to proceed for up to 1000 iterations, with convergence defined by a minimum change threshold of 0.00001. No class-weight correction was applied. The dependent variable was treated as qualitative, and all analyses were conducted using the CHAID method under these pre-pruning constraints, which jointly determined the final tree structure.

#### CHAID accuracy and ten-fold cross-validation

Model performance was assessed using the risk estimate, defined as the proportion of misclassified observations. To evaluate both apparent and generalizable predictive performance and accuracy, risk was calculated under resubstitution (training sample) and 10-fold cross-validation. In the cross-validation procedure, the dataset was randomly partitioned into ten approximately equal subsets; in each iteration, nine subsets were used to train the model and the remaining subset was used for validation, with the process repeated until all subsets had served as validation data once. The cross-validated risk estimate was computed as the average misclassification rate across folds.

Standard errors of the risk estimates were obtained to quantify uncertainty. Agreement between resubstitution and cross-validated risk estimates was used as an indicator of model stability and potential overfitting. Classification accuracy was derived as 1 minus the estimated risk, hence, accuracy was confirmed when resubstitution and cross-validation errors were similar, with the error ratio approaching 1.

Press’s Q statistic was likewise calculated using the formula described above and applied to the 10-fold cross-validation results to assess whether classification accuracy exceeded chance expectation (Navas González 2024).

### Ethics approval, ARRIVE guidelines compliance and consent to participate

All methods and procedures described in this study were performed in accordance with relevant institutional, national, and international guidelines and regulations for the use of animals in scientific research. The study followed the premises described in the Declaration of Helsinki. The Spanish Ministry of Economy and Competitivity through the Royal Decree-Law 53/2013 and its credited entity the Ethics Committee of Animal Experimentation from the University of Córdoba permitted the application of the protocols present in this study as cited in the fifth section of its second article, as the animals assessed were used for credited zootechnical use. This national Decree follows the European Union Directive 2010/63/UE, from the 22nd of September of 2010. This study is reported in accordance with the ARRIVE (Animal Research: Reporting of In Vivo Experiments) guidelines. The work was based exclusively on retrospective analysis of pre-existing records obtained through routine zootechnical management, and no experimental or invasive procedures were performed on live animals. Records were collected following minimal handling practices; therefore, no additional special permissions were required.

## Conclusions

This study demonstrates that cubic regression modelling is a useful exploratory framework for describing age-related variation in semen quality traits in Murciano-Granadina bucks and for identifying genotype-associated patterns in baseline levels and age-related dynamics. By partitioning semen parameters into baseline and higher-order age components, the approach provides a biologically interpretable synthesis of static genotype differences and non-linear ageing trajectories without implying causality. At the αS1-casein locus, genotype-specific semen quality profiles were identified rather than a simple ranking. The EE genotype was associated with higher baseline progressive motility and more favorable membrane-function dynamics, occasionally accompanied by less favorable curvature in volume-related traits. The AE genotype showed relatively favorable baseline sperm concentration and moderate motility, with indications of positive volume dynamics over age. In contrast, AB and BB genotypes displayed intermediate or heterogeneous profiles, often reflecting volume–quality trade-offs, while the frequent BE genotype tended to show lower baseline sperm concentration and less favorable dynamic components, albeit with substantial within-genotype variability. At the κ-casein locus, the AA genotype was most consistently associated with quality-related semen traits, including higher sperm concentration and improved acrosome and membrane integrity, with moderate ejaculate volume. The AB genotype showed generally balanced but more variable profiles, whereas the BB genotype emphasized ejaculate volume at the expense of sperm concentration, motility, and membrane integrity, indicating a clear yield–quality trade-off. Joint αS1–κ genotypes largely reflected additive locus-specific effects. Rare combinations such as EE–AA and AE–AA displayed favorable semen quality profiles but occurred too infrequently for generalization, while more common combinations such as BE–AB represented stable intermediate semen quality. Combinations dominated by BB genotypes (e.g. BB–BB) primarily favored ejaculate volume over sperm quality. All associations should be regarded as descriptive and hypothesis-generating; fertility outcomes were not assessed, and interpretation is constrained by uneven genotype frequencies. Within these limits, cubic regression modelling provides a robust framework for guiding future studies that integrate larger populations and direct fertility endpoints, supporting the broader biological relevance of casein polymorphisms beyond milk traits and their potential consideration in balanced breeding strategies aimed at improving both dairy productivity and male reproductive performance.

## Supplementary Information

Below is the link to the electronic supplementary material.

Supplementary Material 1

Supplementary Material 2

Supplementary Material 3

Supplementary Material 4

Supplementary Material 5

Supplementary Material 6

## Data Availability

The materials have been described in sufficient detail within the paper to allow replication of the study. Data supporting the findings of this work have been included as Supplementary Material in this article and are also available from the corresponding author, F.J.N.G., upon reasonable request.
