# Supplementary material for: Casein genotypes associate with baseline and dynamic regression components of seminal quality in Murciano-Granadina bucks
Source: Sci Rep. 2026 Apr 15;16:17598. doi: 10.1038/s41598-026-43947-1 (PMC13243535; doi:10.1038/s41598-026-43947-1)
Supplement: Supplementary file 6 — Supplementary Material 6 [file 41598_2026_43947_MOESM6_ESM.pdf]

Supplementary Material S1. Implementation of False Discovery rate (FDR) correction in Excel.

False discovery rate (FDR) control was applied to univariate p-values using the Benjamini–Hochberg procedure, implemented post hoc in Microsoft Excel. For each family of related tests, p-values were entered in a single column and verified to be numeric.

Let column **B** contain the original p-values (rows 2 to *n*), where *m* is the total number of tests.

Ranking of p-values
P-values were ranked in ascending order using:

=JERARQUIA(B2;B2:Bn;1)

where the smallest p-value receives rank $i = 1$.

Computation of FDR critical values
For each test, the Benjamini–Hochberg critical value was calculated as:

=(i/m)*0,05

where *i* is the rank of the p-value and *m* is the total number of tests in the family.

Decision rule
A test was considered significant after FDR correction if:

$p \leq (i/m)*0,05$

Computation of FDR-adjusted p-values (q-values)
Adjusted p-values were calculated as:

=(B2*m)/i

To ensure monotonicity, each adjusted p-value was replaced by the minimum of its own value and all adjusted p-values corresponding to higher ranks, yielding a non-decreasing sequence of *q*-values.

All FDR calculations were performed independently for each genotype and test family.

Supplementary Table S1. Mean and standard deviation of the Durbin–Watson statistics for residuals of age-ordered regression models fitted to seminal quality parameters.

Seminal Quality Parameters	Mean	Std. Deviation
Volume	1.74	0.48
Sperm Concentration	1.63	0.56
Total Motility	1.82	0.43
Progressive Motility	1.74	0.52
Abnormal sperm count	1.80	0.47
Endosmosis	1.71	0.56
Total Estimated Volume	1.81	0.54
Total Doses	1.81	0.54
Real added total volume	1.79	0.54
Real Doses	1.78	0.55

Supplementary Table S8. Models and model syntax.

Model	Initials	Model syntax/equation
Linear	LIN	Y = b0 + (b1 * t)
Logarithmic	LOG	Y = b0 + (b1 * ln(t)
Inverse	INV	Y = b0 + (b1/t)
Quadratic	QUA	Y = b0 + (b1 * t) + (b2 * t**2)
Cubic	CUB	Y = b0 + (b1 * t) + (b2 * t**2) + (b3 * t**3)
Power	POW	Y = b0 * (t**b1) or ln(Y) = ln(b0) + (b1 * ln(t))
Compound	COM	Y = b0 * (b1**t) or ln(Y) = ln(b0) + (ln(b1) * t)
S-curve	SCRV	Y = e**(b0 + (b1/t)) or ln(Y) = b0 + (b1/t)
Logistic	LGI	Y = 1/(1/u + (b0 * (b1**t))) or ln(1/y-1/u) = ln (b0) + (ln(b1) * t)
Growth	GRO	Y = e**(b0 + (b1 * t)) or ln(Y) = b0 + (b1 * t)
Exponential	EXP	Y = b0 * (e**(b1 * t)) or ln(Y) = ln(b0) + (b1 * t)

t represents age (time) expressed in month units. Accessed from IBM Corp (2015).

Supplementary Table S6. Detailed and analytical composition of the diet provided to the animals.

Detailed composition	
GMO corn, barley, GMO soybean shell, decorticated soybean meal (47% gross weight), wheat forage flour, sugar beet pulp, wheat, dried residues of corn distillery, partially decorticated sunflower extraction flour, rapeseed flour, cane grassroots, calystic acid sallc, calcium carbonate, bicarbonate, sodium chloride, animal fat.	
Additives, vitamins, provitamins and substances of analogous effect	
Vitamin A	5625.0 UI/Kg
Vitamin D3	1125.0 UI/Kg
Vitamin E	9.0 mg/Kg
Trace element compounds	
Copper as a pentahydrate cubic sulfate	1.0 mg/Kg
Zinc sulfate monohydrate	45.0 mg/Kg
Zinc as a zinc chelate of glycine hydrate	5.0 mg/Kg
Selenium as seleniomethionina produced by Saccharomyces cerevisiae	0.200 mg/Kg
Selenium as sodium selenite	0.230 mg/Kg
Iodine as potassium iodide	1.0 mg/Kg
Manganese as manganese sulfate monohydrate	50.0 mg/Kg
Iron as iron carbonate	30.0 mg/Kg
Cobalt as coated granulated cobalt carbonate (II)	0.5 mg/Kg
Binders and anti-caking agents	
Kieselguhr (purified diatom land)	
Sodium silicate aluminum	
Reducers of feed mycotoxin contamination	
Bentonite	100.0 mg/Kg
Flavouring	
Dried extract of grapes (polyphenols and catechins)	
Mix of natural aromas	
Preservatives	
Ammonium sorbate	
Formic acid	
Analytic composition	
Crude protein	15.28%
Crude oils and fats	3.84%
Crude fiber	9.17%
Crude ash	5.97%
Calcium	0.81%
Phosphorus	0.41%
Sodium	0.44%
Magnesium	0.25%
